# Coherence in Polycrystalline Thin Films of Twisted Molecular Crystals

**DOI:** 10.1021/acs.chemmater.3c02740

**Published:** 2024-01-04

**Authors:** Yongfan Yang, Alexander G. Shtukenberg, Hengyu Zhou, Christian Ruzie, Yves Henri Geerts, Stephanie S. Lee, Bart Kahr

**Affiliations:** † Department of Chemistry and Molecular Design Institute, New York University, New York, New York 10003, United States; ‡ Laboratoire de Chimie des Polymères, Faculté des Sciences, Université Libre de Bruxelles (ULB), Boulevard du Triomphe, CP 206/01, Brussels 1050, Belgium; § International Solvay Institutes of Physics and Chemistry, Université Libre de Bruxelles (ULB), Boulevard du Triomphe, CP 231, Brussels 1050, Belgium

## Abstract

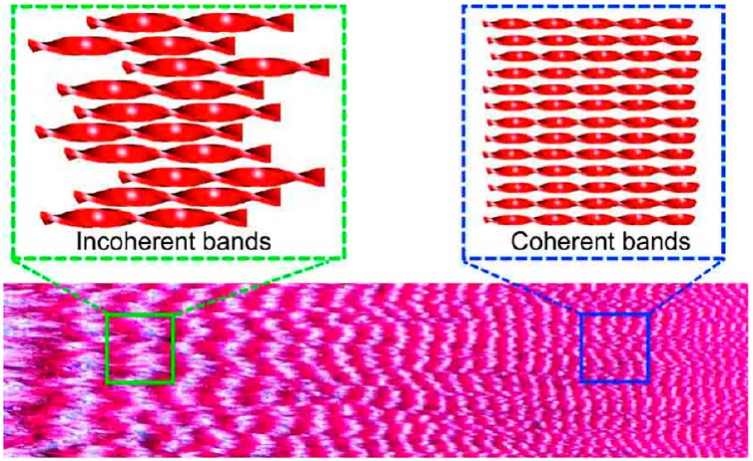

Helicoidal crystallites in rhythmically banded spherulites manifest spectacular optical patterns in small molecules and polymers. It is shown that concentric optical bands indicating crystallographic orientations typically lose coherence (in-phase twisting) with growth from the center of nucleation. Here, coherence is shown to increase as the twist period decreases for seven molecular crystals grown from the melt. This dependence was correlated to crystallite fiber thickness and length, as well as crystallite branching frequency, a parameter that was extracted from scanning electron micrographs, and supported by numerical simulations. Hole mobilities for 2,5-didodecyl-3,6-di(thiophen-2-yl)pyrrolo[3,4-c]pyrrole-1,4(2H,5H)-dione (DPP-C12) measured by using organic field-effect transistors demonstrated that more incoherent boundaries between optical bands in spherulites lead to higher charge transport for films with the same twist period. This was rationalized by combining our growth model with electrodynamic simulations. This work illustrates the emergence of complexity in crystallization processes (spherulite formation) that arises in the extra variable of helicoidal radial twisting. The details of the patterns analyzed here link the added complexity in crystal growth to the electronic and optical properties of the thin films.

## Introduction

Spontaneous pattern formation^1,2^ is one of the great unifying principles that cuts across all the sciences. From the stripes on a zebra to oscillating chemical reactions and the structures of galaxies, often, similar principles can be found in operation. Spectacular patterns often arise in crystallization at high driving forces when many microcrystals compete for space and nutrients. Here, we analyze the details of growth mechanisms, morphologies, and properties in thin films of organic semiconductors that spontaneously organize into complex patterns.

Spherulites are radial polycrystalline aggregates that form from many materials grown under sufficiently high driving forces.^3^ Their formation stems from the high aspect ratio of individual crystallites, noncrystallographic small-angle branching,^4−6^ and geometrical selection processes.^7,8^ Crystallites can be tightly or loosely packed depending on the branching frequency.^3^ Spherulites usually have distinct cores characterized by double-leaf morphologies, arising in the time necessary for a needle-like nucleus to become radial through the mechanism of noncrystallographic branching.^9−12^

The spherulitic crystal growth morphology becomes more complex if crystallites spontaneously twist as they grow.^13−18^ Such so-called banded spherulites display oscillating linear birefringence (LB) as a consequence of continuous changes in crystallite orientation and refractivity.^17,19^ In general, up to one-third of molecular crystals can be made to twist as they grow from the melt.^20−23^ In some families of crystals, such as binary charge transfer complexes (CTCs), the proportion can be greater than one-half.^24^ Due to the anisotropy of crystal cross section shape and branching frequency, twisting substantially complicates fiber organization in spherulites. Previously efforts to account for emergent complexity have relied on microstructural analyses of the 3D arrangements of twisted lamellae in polymer spherulites.^25−27^ A more global analysis emphasized here relies on polarization-dependent optical properties. For instance, circular birefringence measurements of banded spherulites indicate that the adjacent twisted lamellae with anisotropic cross sections cannot grow parallel to each other due to the space constraints in films crystallized between two glass slides. Instead, they bump into one another, misorient, and splay.^28−31^

Fiber organization in banded spherulites is a great example of emerging complexity in prosaic, easily prepared systems, whose evolutions are controlled by a few simple rules. This knowledge may find potential applications in organic semiconductor electronics. It has been well established that grain boundaries act as bottlenecks to charge transport in devices comprising polycrystalline organic active layers.^32,33^ In spherulitic films, the mobility decreases with decreasing spherulite size due to the higher density of grain boundaries.^34−36^ Therefore, researchers have been advancing strategies to increase domain size; single crystals are typically viewed as the gold standard.^37,38^ On the other hand, our group previously studied the effect of crystal twisting on carrier mobility of three tetracyanoethylene-based CTCs^24^ and a monocomponent organic semiconductor BDT^39^ and demonstrated that a greater number of small gaps in twisted crystal films leads to higher mobilities than a fewer number of big gaps in straight crystal films.

Previously^24,39^ we have analyzed how charge mobility depends on the presence and intensity of crystal twisting in films of organic semiconductors. In this work, we focus on the additional complexity of fiber organization and its consequences for charge transport related to the difference in lateral alignment of twisted fibers with the same twist periods. First, we established for seven molecular crystals that the incoherence between bands in optical micrographs increases along spherulite radii and is proportional to the pitch. Then, we proposed a mechanism underlying this correlation and verified it using an analytical model and numerical simulations. Finally, we explored the effect of band boundary incoherence on the linear retardance of coumarin and the charge transport properties of organic semiconductor 2,5-didodecyl-3,6-di(thiophen-2-yl)pyrrolo[3,4-c]pyrrole-1,4(2H,5H)-dione (DPP-C12).^35^

## Results and Discussion

### Band Boundary Incoherence

**Figure 1:**
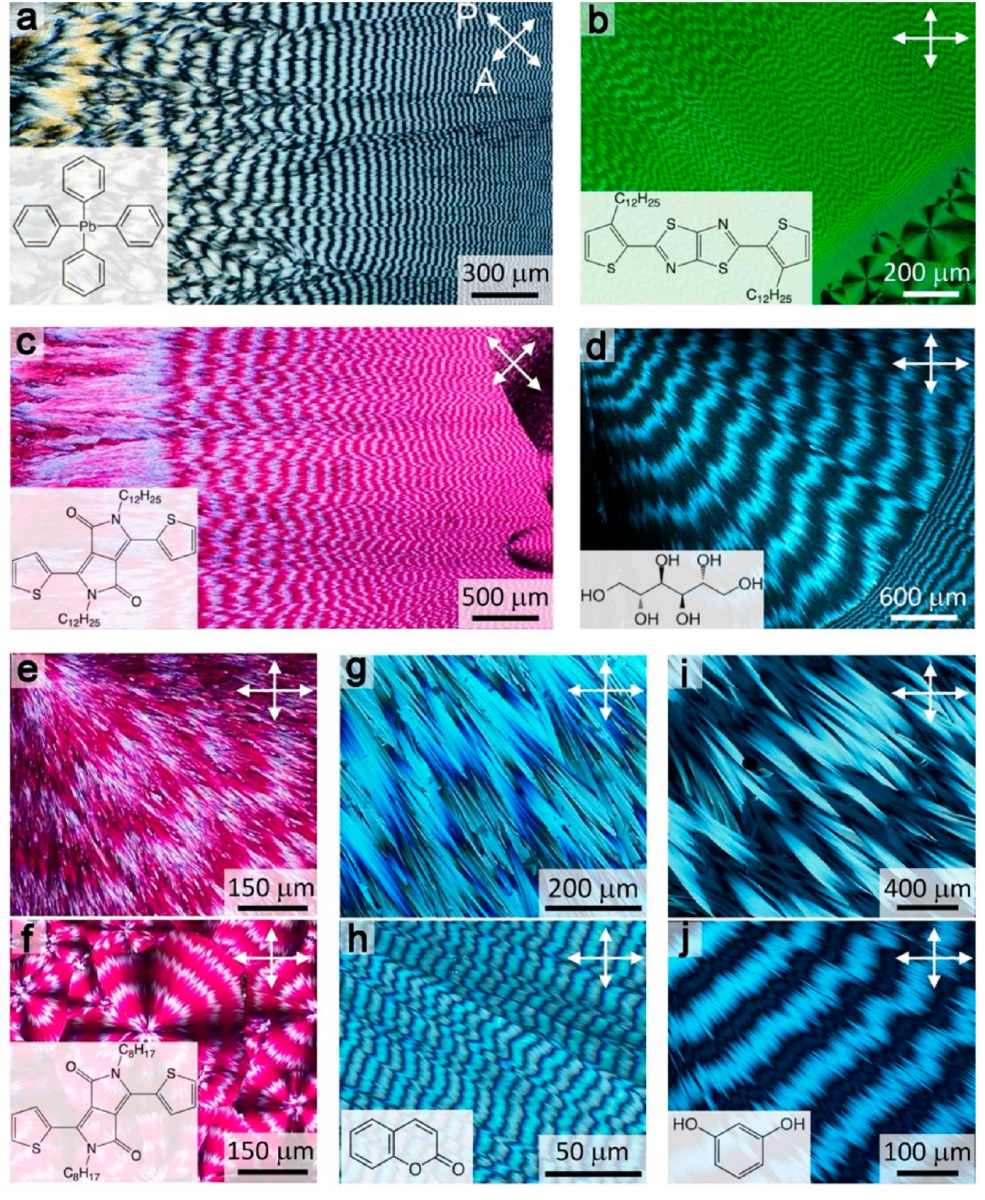
Banded spherulites between crossed polarizers. (a) Tetraphenyl lead with 24.5 wt % of polyvinylpyrrolidone (PVP) grown at *T* = 140–100 °C. (b) 2,5-Bis(3-dodecyl-2-thienyl)-thiazolo[5,4-d]thiazole (BDT) grown at *T* = 65–35 °C. (c) 2,5-Didodecyl-3,6-di(thiophen-2-yl)pyrrolo[3,4-c]pyrrole-1,4(2H,5H)-dione (DPP-C12) grown at *T* = 120–100 °C. (d) δ-Mannitol with 15 wt % PVP grown at *T* = 150 and 100 °C. (e, f) 2,5-Dioctadecyl-3,6-di(thiophen-2-yl)pyrrolo[3,4-c]pyrrole-1,4(2H,5H)-dione (DPP-C8) at *T* = 90 °C (e) and 60 °C (f). (g, h) Coumarin polymorph IV with 20 wt % Canada balsam grown at *T* = 50 °C (g) and 10 °C (h). (i, j) Resorcinol with 25 wt % of tartaric acid grown at *T* = 80 °C (i) and 35 °C (j). (a–d) Pitch decreases continuously with decreasing growth temperature. (e, f), (g, h), and (i, j) Examples of largest and smallest pitches observed for each compound. The directions of the polarizer (P) and analyzer (A) are labeled in the right top corners of each image with double headed arrows.

Banded spherulites show sharp changes in interference colors when viewed between crossed polarizers by virtue of the helicoidal precession of the crystal refractivity. Figure 1a illustrates a banded spherulite of tetraphenyl lead^21^ grown at different temperatures. Twisting period or pitch, *P*, defined as the distance between two successive dark or bright bands (π rotation of a crystal along the growth direction) decreases as the growth temperature decreases. Such behavior is common and, to a significant extent, is related to the formation of finer fibers at higher supercooling.^17,40^ The band boundary appears to be more jagged and more incoherent for larger pitches. A similar trend was also observed here in the other six systems: DPP-C12, 2,5-dioctadecyl-3,6-di(thiophen-2-yl)pyrrolo[3,4-c]pyrrole-1,4(2H,5H)-dione (DPP-C8), 2,5-bis(3-dodecyl-2-thienyl)-thiazolo[5,4-d]thiazole (BDT), δ-mannitol, coumarin, and resorcinol (Figure 1b–g). It was also observed for spherulites of polymers^16,41^ and polymer blends.^42^

**Figure 2:**
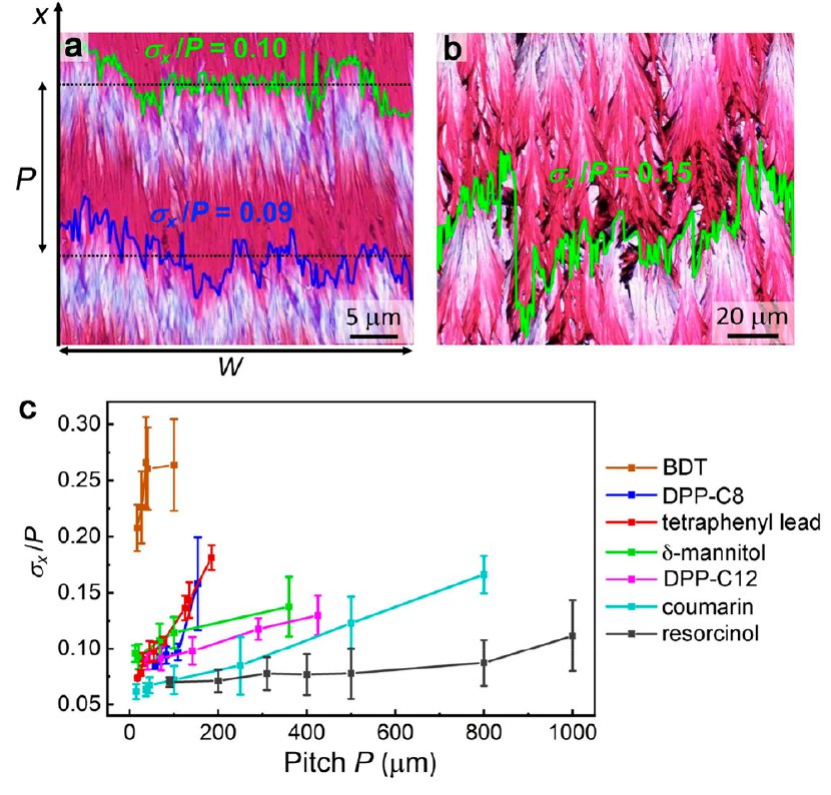
Boundary incoherence dependence on pitch. (a, b) Micrographs of DPP-C12 banded spherulite films between crossed polarizers. Light and dark red regions indicate flat-on and edge-on crystallites, respectively. (a) *P* = 35 μm and (b) *P* = 420 μm combined with the band boundary curves drawn in MATLAB. (c) Dependence of boundary incoherence on the twisting pitch for seven compounds. Error bars indicate the standard deviations of *σ*_*x*_/*P* obtained from measuring five optical images.

Band boundary *x* is identified as the positions corresponding to the same color and, respectively, having the same orientation with respect to illumination direction (i.e., corresponding to a band boundary) and were identified from the images taken with an optical microscope. The area of optical images selected for analysis was 4*P* in length and 2*P* in width to mitigate effects of spherulite fiber divergence. Using a MATLAB script the raw optical images were converted to binary images composed of elements 0 and 1 with a threshold of 0.45. Element 1 was assigned as white for flat-on orientation and element 0 was assigned as a black area for edge-on orientations. The band boundary curves were captured as the transition region from edge-on to flat-on orientations with a specific gray scale value of 0.45 (Figures 2a and S1). The boundary incoherence was defined as the standard deviation of the boundary position *x* along the growth direction normalized by the pitch, *σ*_*x*_/*P*. *σ*_*x*_/*P* = 0 when the fibers are in-phase (Figure S2a), and increases as fibers go out of phase (Figure S2b). The relative boundary incoherence defined this way compares incoherences measured for spherulites with very different pitches.

Examples of optical micrographs of DPP-C12 films with pitch *P* = 35 and 420 μm are shown in Figure 2a,b, along with the respective boundary curves drawn in MATLAB. DPP-C12 banded spherulites were grown from the melt (*T*_m_ = 125 °C) between 100 and 122.5 °C (Figures S3 and S4). As the temperature decreases, the pitch decreases. Below 100 °C high nucleation densities limit spherulite sizes, and banding disappears. Boundary incoherence *σ*_*x*_/*P* increased with increasing pitch *P* (Figure 2c). The pattern width *W* was set between *P* to 1.5*P* to avoid the spherulite curvature and approximate a straight growth front.

A comparable dependence of incoherence on *P* was observed for all of the other compounds: DPP-C8, BDT, tetraphenyl lead, δ-mannitol, coumarin, and resorcinol (Figure 2c). Among them, tetraphenyl lead and DPP-C8 exhibit the strongest *σ*_*x*_/*P* vs *P* dependencies. Coumarin and resorcinol show wider *P* ranges, while the dependence of *σ*_*x*_/*P* on *P* is weaker. BDT follows the same trend but has much higher *σ*_*x*_/*P* due to concurrent twisting and bending (Figure S5).^39^

### Pitch-Dependent Boundary Incoherence

**Figure 3:**
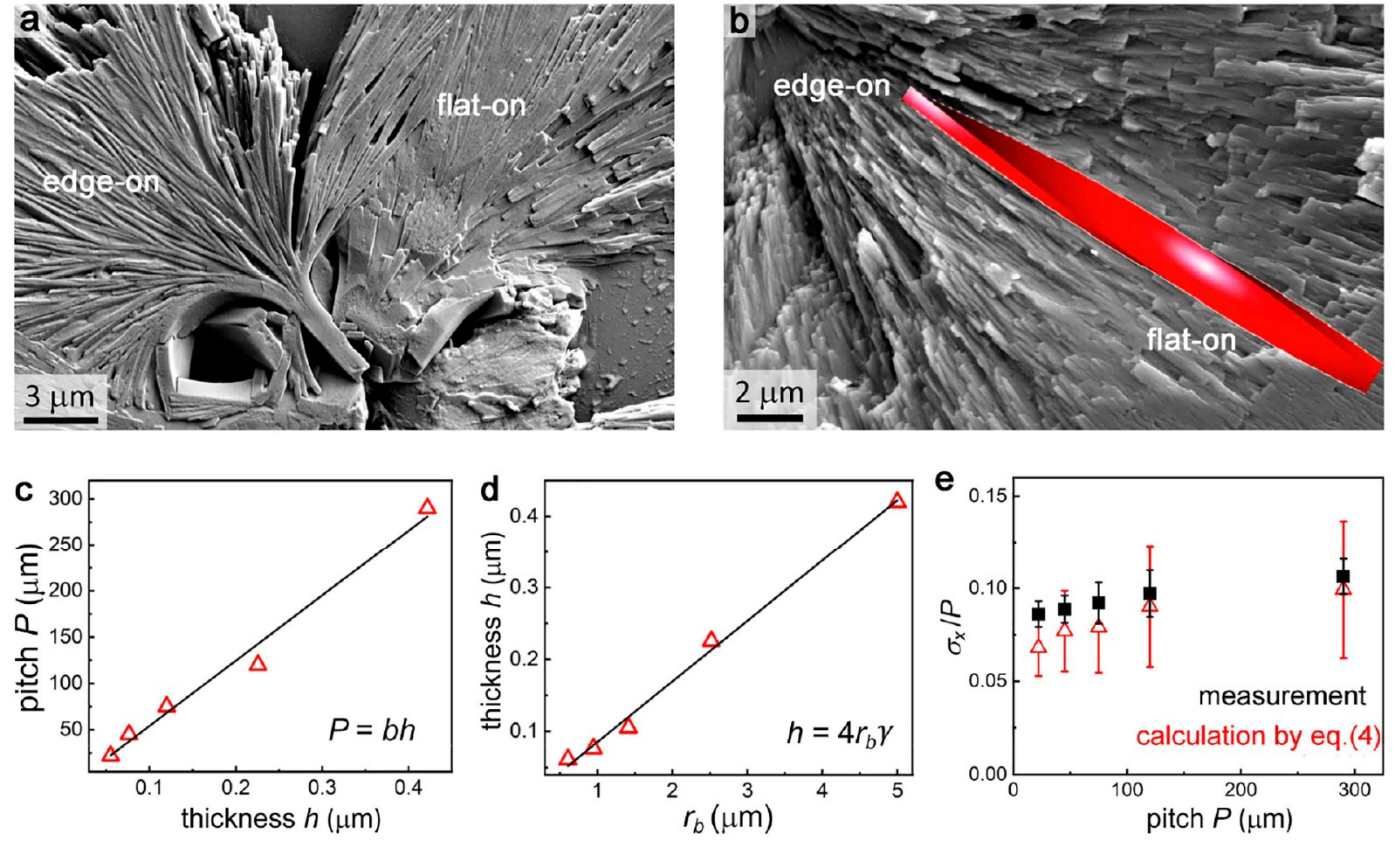
DPP-C12 spherulites and their geometrical parameters. (a, b) SEM images of twisted crystals showing flat-on and edge-on orientations and transition regions between them. (b) Overlayed red helicoid highlights the right-handedness of fibers. (c) Correlation between experimentally measured pitch and fiber thickness. (d) Correlation between the fiber thickness and the distance between two successive branching events, both measured from SEMs. (e) Experimentally determined (black squares) *σ*_*x*_/*P* versus calculated values using equation 4 (red triangles).

At the center of a spherulite, all fibers branch from the same nucleus and are in phase (Figure 3a, Figure S6). As observed in SEM images (e.g., Figure S7 for DPP-C12 but similar observations were made for other materials), individual fibers have slightly different thicknesses *h*, which can be described by a Gaussian distribution with a dispersion *σ*_*h*_ (Figure S10). It has been well established for most of crystals with twisted fibers^17,40^ that pitch *P* is proportional to *h* as equation 1
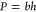
1where *b* is a proportionality constant (Figure 3c). Linear relationships between *h* and *P* were also obtained for five other compounds, DPP-C8, BDT, coumarin, mannitol, and resorcinol (Figure S9), except for tetraphenyl lead, whose individual fibers cannot be distinguished by SEM.

We assume that pitch variations for a spherulite forming at constant conditions are solely related to the thickness variations. Pitch variance  can be obtained from thickness variance  via equation 2 using properties of variance and equation 1.
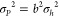
2If every fiber maintains the same thickness throughout the whole spherulite, position variance after the first band (*N* = 1) will be equal to the pitch variance, . For subsequent bands *N* > 1, accumulation of band variances yields σ_*x*_^2^ = *N*σ_*P*_^2^. In reality, fibers undergo multiple branching and collision events and usually do not extend for the length of one pitch. The average length of the fiber can be defined as *l* = *P*/*n*, where *n* is a number of fibers comprising the length of one *P*. Renucleation of new fibers with different thicknesses decreases incoherence due to the averaging of the distance required to make a 180° rotation of the fiber. Thus, for the first band in the spherulite, the boundary variance gets smaller as *n* grows: . Incorporation of a fiber discontinuity for a band *N* ≥ 1 yields equation 3:
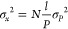
3This equation can be converted to a more testable form using equations 1 and 2 as equation 4:
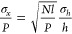
4To verify equation 4, *l*, *h*, and *σ*_*h*_ were measured from SEM images while *P* and *N* were obtained from optical micrographs. *σ*_*x*_/*P* calculated from equation 4 agreed well with *σ*_*x*_/*P* extracted directly from optical micrographs for DPP-C12 (Figure 3e). The error bars for experimentally determined values were obtained as standard deviations of *σ*_*x*_/*P* measured for several different areas of the same sample. The error bars for the values obtained using equation 4 were estimated using experimentally determined standard deviations for *P*, *l*, and *h*. One can see that measured and calculated *σ*_*x*_/*P* show the same trend, and their values agree with one another within their standard deviations. Good agreement was also obtained for δ-mannitol (Figure S11). Analysis for other compounds was precluded by the difficulty of measuring fiber lengths.

Average fiber thickness *h* is predicted to be proportional to the distance between successive branching events *r*_b_ as *h* = 4*r*_b_γ,^3^ where γ is the average misorientation angle. This prediction is confirmed for DPP-C12 crystals in Figure 3d and for coumarin in Figure S8. γ = 1.27° for DPP-C12 and 1.07° for coumarin as calculated from experimentally measured values of *h* and *r*_b_. This estimate is consistent with the literature data for other compounds, γ = 0.2°–0.9°.^3^ The ratio *σ*_*h*_/*h* is consistent for all materials and varies in the range of 0.05–0.21 with the majority of values being in the range 0.09–0.16. Since *l* is generally proportional to *P* and *σ*_*h*_ is proportional to *h*, combining equations 1–3, one can expect *σ*_*x*_/*P* ≈ const. The incoherence increase with pitch results from less aggressive branching at higher temperature. Under these conditions the branching frequency () is lower, fiber thickness and pitch are larger, and spherulites are less compact with less cooperative fibers.

### Growth Morphology Simulations

**Figure 4:**
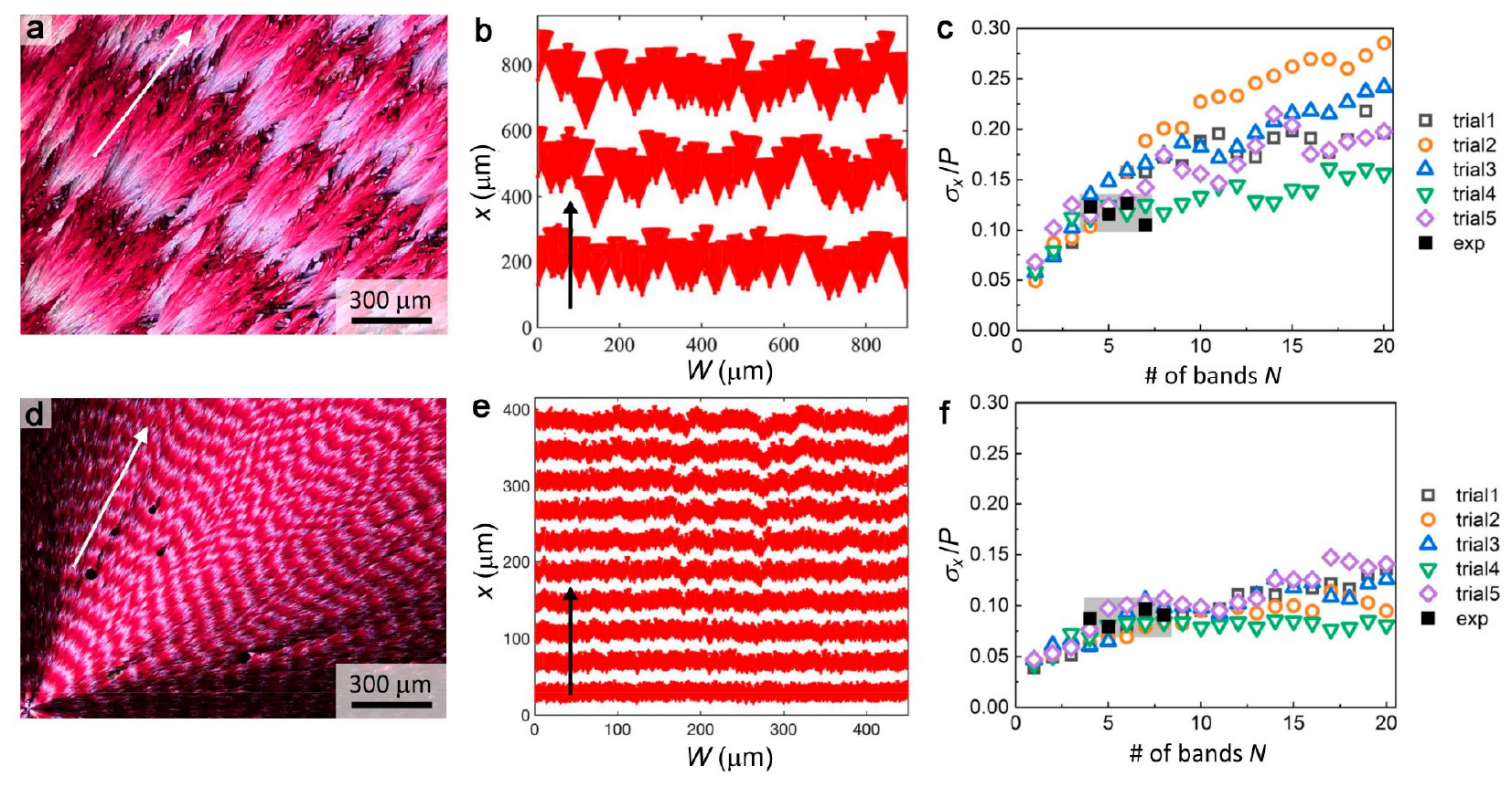
Comparison of optical micrographs and simulated patterns for DPP-C12 films. (a–c) Pitch *P* = 300 μm grown at 112 °C. (d–f) Pitch *P* = 45 μm grown at 105 °C. (a, d) Polarized optical images. (b, e) Simulated patterns. The white arrows in (a) and (d) and black arrows in (b) and (e) indicate the crystal growth direction. (c, f) Simulated boundary incoherence as a function of band number (pattern width *W* = 2*P*). Open symbols, simulation results; solid symbols, experimental measurements.

The aforementioned mechanism assumes that the branching frequency is constant for the same pitch. The fiber cross sections, however, are not isometric but rectangular with flat-on and edge-on orientations (Figure 3a,b), which have different branching frequencies. Video S1 demonstrates that light red, flat-on oriented crystal fibers form a compact growth front due to the higher branching frequency. In comparison, dark red edge-on fibers form a loosely organized growth front because of the weaker branching frequency. The extra spaces are filled later by randomly oriented fibers resulting in a “flower” morphology, where the “stem” indicates the edge-on orientations and “petal” represents the flat-on orientation (Figure 4a,d). This morphology is common for banded spherulites and was also observed in BDT and δ-mannitol (Figure S12).

Different branching frequencies for flat-on and edge-on orientations (Figure S13) were accounted for in simulations (“*Numerical simulation of fiber organization in banded spherulite*” in the Supporting Information). The observed pattern is controlled by *h*, *σ*_*h*_, *P*, and the opening angle 2θ for a flat-on orientation (Figure S14) and by the pattern size (width *W* × band number *N*).

The patterns simulated for DPP-C12 using experimentally measured *h*, *σ*_*h*_, and 2θ for *P* = 300 μm (Figure 4b) and *P* = 45 μm (Figure 4e) closely resemble the optical images (Figure 4a,d). The edge-on orientations are shown in white, while the compact flat-on orientations are in red.

Five independent simulations were performed for DPP-C12 films with *P* = 300 μm (Figure 4c). As the band number increased in each case, the boundary incoherence increased. The simulated incoherence agrees with the experimental value for the same pattern width *W* = 2*P* and the distance from the spherulite center (4 < *N* < 8). A similar trend was observed for *P* = 45, 75, and 120 μm (Figure 4f and Figure S15).

An increase in pattern width provides better consistency in the boundary incoherence among different trials (Figure S16). Boundary incoherence is comparable for the first ten bands, after which the difference between trials emerges. When 2θ approaches 0, fibers do not collide with their neighbors and *σ*_*x*_/*P* ∝  as predicted by equation 1 (Figure S17).

### Boundary Incoherence and Linear Retardance

**Figure 5:**
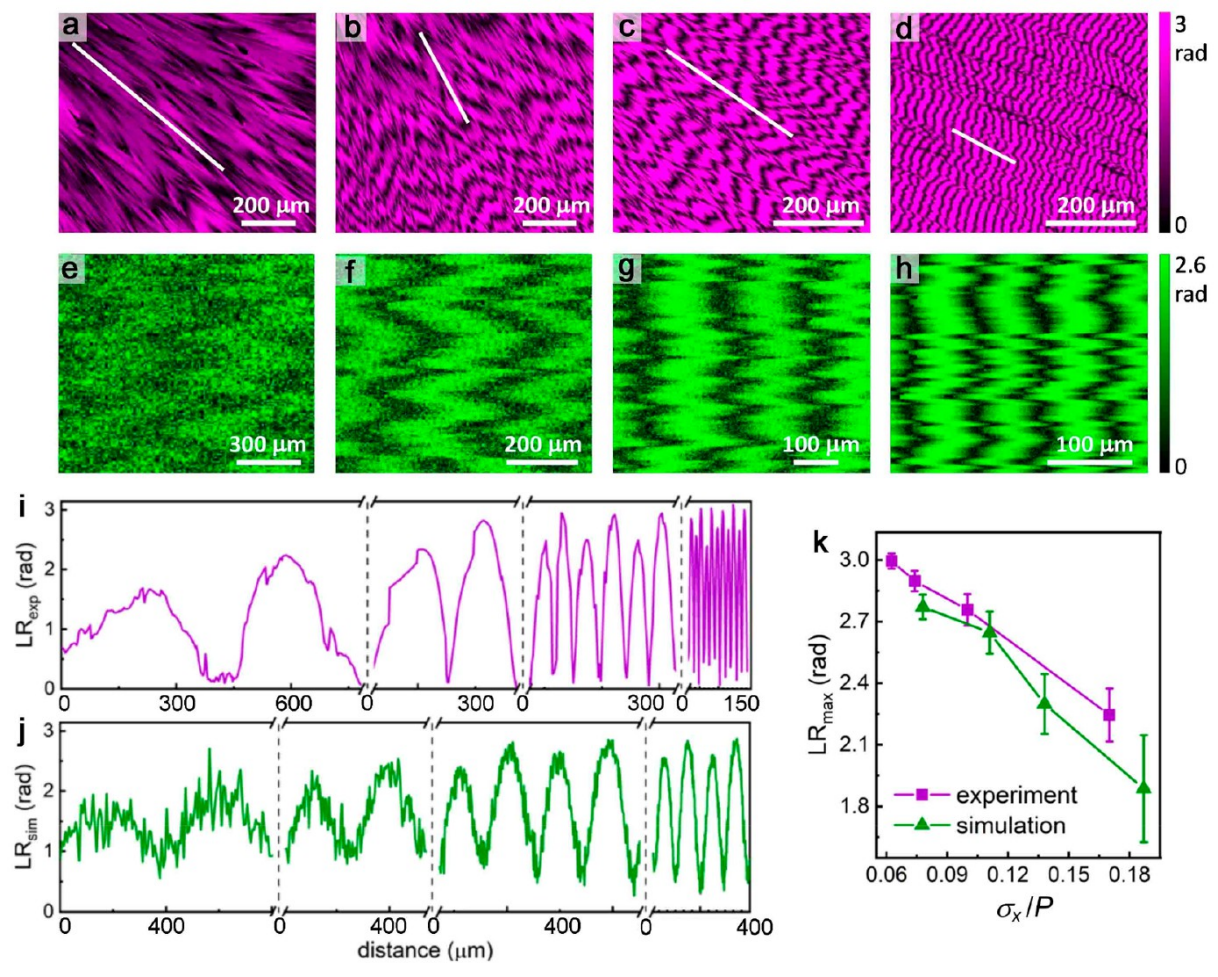
Linear retardance of the twisted coumarin crystal based on experiment and simulation. (a–d) Experimental |LR| false color micrographs for twisted crystals with different *P* and *σ*_*x*_/*P* measured at λ = 550 nm. (a) *P* = 800 μm and *σ*_*x*_/*P* = 0.17. (b) *P* = 360 μm and *σ*_*x*_/*P* = 0.10. (c) *P* = 125 μm and *σ*_*x*_/*P* = 0.074. (d) *P* = 28 μm and *σ*_*x*_/*P* = 0.063. (e–h) Simulated |LR| false color micrographs. (e) *P* = 980 μm and *σ*_*x*_/*P* = 0.19. (f) *P* = 600 μm and *σ*_*x*_/*P* = 0.14. (g) *P* = 400 μm and *σ*_*x*_/*P* = 0.11. (h) *P* = 150 μm and *σ*_*x*_/*P* = 0.078. (i, j) Extracted |LR| along the fiber growth direction depicted by white lines in (a)–(d) and the whole horizontal range in (e)–(h), respectively. (k) Comparison of maximum |LR| from experiment and simulation for twisted coumarin crystals with varied *σ*_*x*_/*P*.

Linear retardance (LR) is the path difference gained by orthogonal polarization modes vibrating in anisotropic crystals that is proportional to the intrinsic difference in refractive activity, as well as the path length. Woo et al. observed that spherulites with incoherent twisted fibers have lower LR compared to spherulites with coherent fibers.^25,26^ In other words, coherent fibers better approximate the LR of a single crystal. Here, we measured LR for twisted coumarin crystals with different boundary incoherence using Mueller matrix microscopy (Supporting Information). Figure 5a–d shows |LR| false color micrographs of twisted crystals with *P* decreasing from 800 to 28 μm and *σ*_*x*_/*P* decreasing from 0.17 to 0.063. The film thickness determined by atomic force microscopy was 2.7–3.0 μm. As predicted, the maximum |LR| increases as *σ*_*x*_/*P* decreases (Figure 5i,k).

In our previous observations,^28^ optical properties of banded spherulites are influenced by superimposition of twisted fibers in the *z* direction (parallel to incident light). Here, we simulated |LR| of spherulites considering boundary incoherence along the *z* axis and assuming *σ*_*z*_ = *σ*_*x*_. Simulated LR for the patterns in Figure 5e–h is consistent with experimental results. See the “*Linear Retardance Simulation*” in the Supporting Information.

### Boundary Incoherence and OFET Hole Mobilities

**Figure 6:**
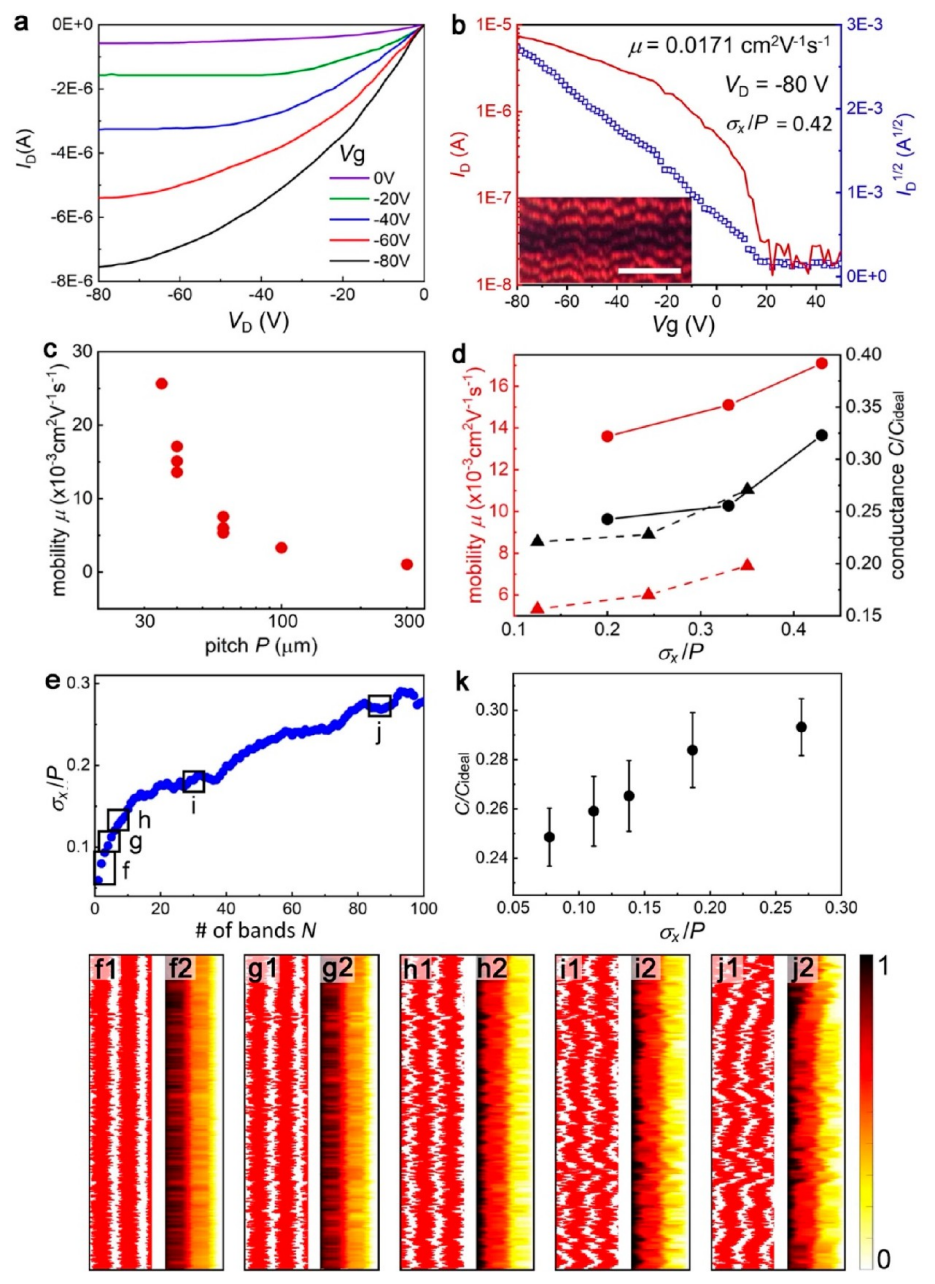
OFET measurement of twisted DPP-C12 crystals and conductance calculation based on the simulated patterns. Output curves (a) and transfer curves (b) of a representative DPP-C12 OFET with *P* = 40 μm, boundary incoherence *σ*_*x*_/*P* = 0.42, and hole mobility μ = 0.0171 cm^2^ V^–1^ s^–1^. The scale bar of the inset in (b) is 200 μm, and channel width *W* = 500 μm. Gate dielectric capacitance per unit area *C*_*i*_ = 1.06 × 10^–8^F·cm^–2^. (c) OFET measured hole mobilities as a function of pitch. (d) OFET measured hole mobility and model simulated conductance as a function of the boundary incoherence of twisted crystals with *P* = 40 μm (solid lines) and *P* = 60 μm (dashed lines). (e) Simulated incoherence as a function of band number *N* for DPP-C12 films with *P* = 45 μm. (f1–j1) Corresponding simulated patterns with *σ*_*x*_/*P* of 0.078 (f1), 0.111 (g1), 0.138 (h1), 0.187 (i1), and 0.270 (j1). (f2–j2) Calculated electrical potential distribution for hole transport under the applied electrical potential Δ*U* = 1 V from the left to the right side for each simulated pattern. The pattern width *W* = 500 μm and length *L* = 100 μm. (k) Relative conductance for hole transport as a function of incoherence. All data points are shown along with the calculated mean values and standard deviations based on six different patterns for each *σ*_*x*_/*P*. Coefficients α = 0.1 and β = 1.

DPP-C12 was reported to have the hole mobilities of 1.32 × 10^–2^ cm^2^ V^–1^ s^–1^ for spin-coated thin films.^35^ Here, bottom-gate bottom-contact organic field effect transistors (OFETs) were fabricated to measure the carrier mobilities of melt-grown twisted DPP-C12 films (Figure S18a). The channel length from source to drain *L* = 100 μm and the width *W* = 500 μm. The pitch was controlled by growth temperature, and samples with different pitches were grown on different OFET substrates. The boundary incoherence was controlled by preparing devices on bands having different distances from the center of spherulite (incoherence varies because of differences in *N*). In Figure 6c, points indicate measurements made on different OFET devices. The total number of devices is only ten because of difficulties of growing the required morphology between electrodes and difficulties in making good electric contacts; however, the data is self-consistent, indicating good reproducibility of the measurements. See “*OFET Fabrication and Electrical Characterization*” in the Supporting Information.

Figure 6a displays output curves for a representative device with *P* = 40 μm. Typically, saturation occurs at source–drain *V*_D_ = −80 V. This bias was applied to measure the corresponding transfer characteristics by sweeping the gate voltage *V*_G_ from −80 to 50 V (Figure 6b). The hole mobility was calculated as 0.0171 cm^2^ V^–1^ s^–1^ based on the slope of  versus *V*_G_. Hole mobilities measured using the same device configuration increased by 25 times as pitch decreased from *P* = 300 μm to *P* = 35 μm (Figure 6c). This trend is consistent with other organic semiconductors, showing higher mobilities for smaller-pitched twisted crystals.^24,39,43,44^

Hole mobilities were measured for three different devices with the same pitch *P* = 40 μm but different *σ*_*x*_/*P* values measured at different band numbers *N* (Figure 6b and Figure S18). The hole mobility is 1.3 times larger for the films with the largest *σ*_*x*_/*P* = 0.42 compared to those with the smallest *σ*_*x*_/*P* = 0.20 (Figure 6d). A similar trend was observed in crystals with larger pitch *P* = 60 μm; hole mobilities were 1.4 times larger in films where *σ*_*x*_/*P* = 0.35, compared to those with 0.13 (Figure 6d, Figure S19).

### Boundary Incoherence and Simulated Conductance

The effect of boundary incoherence on charge transport was also analyzed using electrodynamic simulations,^39^ see “*Effective Conductivity Calculation of a Resistor Network*” in the Supporting Information. DPP-C12 patterns with the same *P* = 45 μm but various *σ*_*x*_/*P* values were simulated as outlined above in section Growth Morphology Simulations (Figure 6f–j). The pattern size was 100 μm × 500 μm, comparable to OFET channel dimensions.

Conductance values for resistors parallel and perpendicular to the fiber elongation direction were selected to account for anisotropic carrier mobilities obtained from density functional theory (DFT) calculations (Figure S23). The crystal orientation and growth direction were determined by 2D XRD patterns (see “*Crystal Orientation Determination of DPP-C12*” in the Supporting Information). Along the crystal growth direction ⟨010⟩, the conductance for flat-on oriented fibers was assigned as the elements of mobility tensor μ_22_ (Table S2), while the value for edge-on fibers was *αμ*_22_, where the coefficient α < 1 accounts for the poorer charge transport at edge-on orientations where the fibers are loosely packed and more discontinuous along the growth direction (Video S1). The conductance for flat-on orientations perpendicular to fiber elongation ⟨001⟩ is *βμ*_33_, where the coefficient β describes the hindering effect of boundaries between fibers when the electric field is perpendicular to the growth direction. For edge-on orientations, the conductance perpendicular to fiber elongation is μ_11_ = 0 (Figure S23c).

Figure 6f–j shows the calculated electric potential distribution for hole transport through films with the coefficients α = 0.1 and β = 1. The potential is highest at the left edge and lowest at the right, and the potential drops sharply at the boundaries. The relative conductance *C*/*C*_ideal_ is compared for simulated patterns with the same *P* but different *σ*_*x*_/*P*. The ideal conductance *C*_ideal_ = 5 μ_22_ is the calculated conductance for a perfect film with only flat-on oriented fibers (Figure S25).

Six trials using each of the conditions in Figure 6f–j were used to extract parameters listed in Figure 6k and Table S3. *C*/*C*_ideal_ increases as *σ*_*x*_/*P* increases, showing 20% larger *C*/*C*_ideal_ for patterns with *σ*_*x*_/*P* = 0.270 vs *σ*_*x*_/*P* = 0.078. The scattering of *C*/*C*_ideal_ values results from different ratios of areas with flat-on and edge-on orientations, as illustrated for the patterns with *σ*_*x*_/*P* = 0.187 (Figure S26). *C*/*C*_ideal_ increases for larger ratios of flat-on orientations, while larger *σ*_*x*_/*P* shows larger *C*/*C*_ideal_ for the patterns with the same ratios (Figure S27).

Further insight was obtained by varying the coefficients α and β. When α = 0, there is no conductance for edge-on oriented fibers along the growth direction. For the pattern with the smallest *σ*_*x*_/*P* = 0.078, edge-on regions can completely block charge transport, leading to *C*/*C*_ideal_ ≈ 0 (Figure S28a,b). As *σ*_*x*_/*P* increases, charge transport improves because of more contacts between adjacent flat-on bands. Progressing from Figure S28a,b through Figure S28i,j, the incoherence grows by a factor of 4, from *σ*_*x*_/*P* = 0.078 to 0.270, while the conductance grows by 5 orders of magnitude from *C*/*C*_ideal_ = 1.73 × 10^–6^ to 1.17 × 10^–1^. When α increases, *C*/*C*_ideal_ increases but its dependence on *σ*_*x*_/*P* is weaker. At the other extreme, α = 1, all patterns have *C*/*C*_ideal_ = 1 (Figure S29a). Because smaller α values correspond to larger differences of the conductance between flat-on and edge-on oriented regions, the effect of boundary incoherence on the conductance becomes stronger. Similarly, *C*/*C*_ideal_ increases as β increases, while the enhancement of conductance by the boundary incoherence is lower (Figure S29b). Lower β corresponds to more isolated fibers with weaker cooperativity, providing a weaker effect of boundary incoherence on conductance.

Optical micrographs of DPP-C12 crystals grown on OFETs were also applied to the electrodynamic simulations. Figures S30 and S31 display optical images of twisted crystals combined with the boundary curves and the simulated electrical potential distributions using α = 0.1 and β = 1 with *P* = 40 and 60 μm, respectively. The slope between *C*/*C*_ideal_ and *σ*_*x*_/*P* is consistent with the relationship between OFET mobilities μ and *σ*_*x*_/*P* (Figure 5d). The intercepts are different since *C*/*C*_ideal_ simulations only evaluate the effect of *σ*_*x*_/*P* while mobilities depend on both *σ*_*x*_/*P* and *P*.

## Conclusions

As evidenced for seven molecular crystals grown from the melt (DPP-C12, DPP-C8, BDT, δ-mannitol, coumarin, tetraphenyl lead, and resorcinol), banded spherulites with larger twist periods (pitches) are characterized by more incoherent (less in-phase) organization. This trend reflects lower branching frequency and a weaker geometrical selection process for larger pitches that leads to a larger variance of crystallite fiber thickness and pitches. The phenomenological analysis was supported by numerical simulations. OFET measurements on DPP-C12 organic semiconductor films show that crystals with the same pitches have higher hole mobility if the fiber organization is more incoherent. For twisted crystal with pitch *P* = 40 um, the hole mobility is 1.3 times larger for the films with the largest *σ*_*x*_/*P* = 0.42 compared to those with the smallest *σ*_*x*_/*P* = 0.20. A similar trend was observed in crystals with larger pitch *P* = 60 μm; hole mobilities were 1.4 times larger in films where *σ*_*x*_/*P* = 0.35, compared to those with 0.13. This correlation was further verified by DFT calculations of anisotropic mobilities combined with electrodynamic simulations. This work identifies and accounts for the fine structure of complex, self-organized polycrystalline patterns and underscores its importance for charge transport in organic semiconductor films.

## Experimental Section

### Melt Crystallization

Melt crystallization was performed by sandwiching several milligrams of a crystalline powder between two coverslips. The material was melted on a hot plate at around 10 °C above melting point to form films with the thickness of 1–2 μm. Generally, the melt was subjected to the target crystallization temperature in a Mettler Toledo hot stage for 5 s. Crystallization started at least 10 s after putting the melted materials at the growth temperature: more specifically, tetraphenyl lead with 24.5 wt % of polyvinylpyrrolidone (PVP) melt at 230 °C and grown at *T* = 140–100 °C with smaller pitches, 2,5-bis(3-dodecyl-2-thienyl)-thiazolo[5,4-d]thiazole BDT melt at 75 °C and grown at *T* = 65–35 °C, 2,5-didodecyl-3,6-di(thiophen-2-yl)pyrrolo[3,4-c]pyrrole-1,4(2H,5H)-dione DPP-C12 melt at 125 °C and grown at *T* = 120–100 °C, δ-mannitol with 15 wt % PVP melt at 165 °C and grown at *T* = 150–100 °C, 2,5-dioctadecyl-3,6-di(thiophen-2-yl)pyrrolo[3,4-c]pyrrole-1,4(2H,5H)-dione DPP-C8 melt at 145 °C and grown at *T* = 90–60 °C, coumarin polymorph IV with 20 wt % Canada balsam melt at 75 °C and grown at *T* = 50–10 °C, and resorcinol with 25 wt % tartaric acid melt at 110 °C and grown at *T* = 80–35 °C.

### Powder X-ray Diffraction

X-ray microdiffraction (μ-XRD) was performed using a Bruker D8 Discover General Area Detector Diffraction System (GADDS) equipped with a VÅNTEC-2000 two-dimensional (2D) detector and a sealed Cu Kα source (λ = 1.54178 Å). The X-ray beam was monochromated with a graphite crystal and collimated with a 0.5 mm capillary collimator (MONOCAP). The sample was loaded on a silicon chip for data acquisition, and the sample-to-detector distance was 145 mm.

### Polarized Absorption Spectra

Optical absorption by the films was measured using a CRAIC Technologies 508 PV microscope spectrophotometer in the range 300–1000 nm equipped with a polarizer. Aperture sizes were chosen for tightly and loosely twisted crystals to maintain the ratio of aperture size to pitch. Crystallite orientations vary within the aperture by ca. 10°.

### Band Boundary Curve from Optical Images

To measure the boundary incoherence in banded spherulites, first, positions corresponding to the same color and, respectively, having the same orientation with respect to illumination direction (i.e., corresponding to a band boundary) were identified from the images taken with an optical microscope. The raw optical images were converted to binary images composed of elements 0 and 1 using a threshold of 0.45. Element 1 was assigned as white for flat-on orientation, and element 0 was assigned as black area for edge-orientation. The band boundary curves were captured as the transition region from edge-on to flat-on orientations with a specific gray scale value of 0.45 (Figure S1).

### Scanning Electron Microscopy (SEM)

Samples were mounted on conductive carbon tape, fastened to aluminum holders, and coated with 5 nm of gold. The images were recorded with a Carl Zeiss MERLIN field emission scanning electron microscope using standard detectors: Everhart-Thornley type and annular secondary electrons with an acceleration voltage of 5 kV and current 110 pA.

### Numerical Simulations of Fiber Organization in Banded Spherulites

The spherulite was assumed to be formed by extreme fiber orientations: flat-on with a high branching rate and edge-on with a low branching rate. Edge-on fibers change orientations to become flat-on randomly according to a Gaussian distribution with an average edge-on segment length equal to *P̅*/2 with the average pitch *P̅* and dispersion *σ*_*P*_. The length of the *i*^th^ fiber (*i* = 1, 2, ..., *M* – total number of fibers used in simulation, *M* ∼ 500) with an edge-on orientation calculated as half of the pitch *l*_*i*_ = *P*_*i*_/2 = *bh*_*i*_/2 assuming that thickness *h*_*i*_ is distributed normally with a mean *h̅* and dispersion *σ*_*h*_. Edge-on fibers are usually loosely packed with the distance of *d*_*i*_ calculated based on the average fiber separation obtained from SEM images.

Fibers with flat-on orientations have a higher branching rate, resulting in compact sector-like shapes (“petals”), with a radius *P̅*/2 and a constant opening angle 2θ. Once a new petal advances by *P̅*/2, the flat-on fibers become edge-on, and the cycle repeats. Neighboring petals typically collide so that the resulting pattern is determined by a geometrical selection process. The simulations are detailed in the Supporting Information.

### Mueller Matrix Microscopy

Crystal optical properties were established with a Mueller matrix microscope, described previously,^28,45,46^ using a xenon arm lamp. Linear retardance LR = 2π(η_0°_ – η_90°_)*L*/λ represents a phase difference between orthogonally plane polarized light (expressed in rad) and is proportional to the path length, *L*. Here η_0°_ and η_90°_ are the refractive indices for orthogonal polarizations, and λ = 550 nm is wavelength of light.

### Atomic Force Microscopy (AFM)

AFM measurements were performed in contact mode using a Bruker Multimode 8 AFM instrument equipped with Bruker DNP-10 Si_3_N_4_ tips on silicon nitride cantilevers with a spring constant of 0.12 N/m (triangular tip B, 205 μm length, and 40 μm width). The images were analyzed by the software package Gwyddion.^47^

### Linear Retardance Simulation

The simulation of linear retardance used a model developed to compute the optical properties of twisted spherulites using MATLAB.^28,31,46^ This model computed the Mueller matrix of materials based on their electric permittivity tensor and misorientation of all fibers along the light pathway. This algorithm calculated the optical properties of a stack of fibers along the *z*-direction normal to the plane of a glass slide in the *xy-*plane, hence, band boundary incoherence along the *z*-direction was considered a factor to influence linear retardance. Boundary incoherence along the *z*-direction (*σ*_*z*_/*P*) was taken as incoherence along the *x*-direction (*σ*_*x*_/*P*) obtained from optical images. The pitch *P* was obtained from optical images and the fiber thickness *h* was measured from SEM images. The Matlab program can be found on Github: https://github.com/hz2134-chem/KahrWardGroup/tree/main/Band_Coherence_Optics.

### Organic Field Effect Transistor (OFET) Fabrication and Electrical Characterization

For OFETs, a bottom-gate, bottom-contact structure was used, where the bottom gate consisted of highly doped silicon with 300 nm of thermally grown SiO_2_ as the gate dielectric (Figure S18a). Source and drain contacts were patterned by photolithography and deposited by ε-beam evaporation (5 nm Cr followed by 50 nm Au) and a channel length of 100 μm. The channel is 100 μm in length and 500 μm in width. Prior to vacuum deposition, the substrates were cleaned in an ultrasonic bath for 15 min in DI water, acetone, and isopropyl alcohol. The twisted crystals were grown from the melt on the devices and were measured by a Keithley 2636B system source meter. OFET measurements were performed at room temperature.

### Density Functional Theory (DFT) Calculations

The calculation of transfer integrals, reorganization energy, and mobility parameters were implemented using the DFT with the B3LYP functional and the 6-31G(d) basis set, using the Gaussian 16 package.

### Effective Conductivity Calculation of a Resistor Network

The sample surface was considered as a rectangular grid with all nodes connected by resistors (Figure S24). Based on Kirchhoff’s and Ohm’s laws, the total current flowing in and out of each node *i* is
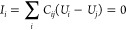
5where *C*_*ij*_ is the conductance of the resistor connecting node *i* and surrounding node *j*, and *U*_*i*_ and *U*_*j*_ are voltages at nodes *i* and *j*, respectively. The total conductance of the network, *C*_tot_, can be expressed as equation 6 for the difference in applied electrical voltages Δ*U* = 1 V
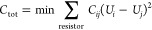
6See details in the Supporting Information.

## Data Availability

Accession code CCDC 2267758 contains the supporting crystallographic data for this paper.

## Supporting Information Available

Details of instrumental methods, OFET mobility measurements, and computational details (PDF)
Growth of twisted DPP-C12 crystals from the melt (MP4)
Crystallographic information file (CIF)

## Supplementary Material

cm3c02740_si_002.mp4

cm3c02740_si_003.cif
