# Supplementary material for: Coherence in Polycrystalline Thin Films of Twisted Molecular Crystals
Source: Chem Mater. 2024 Jan 4;36(2):881–91. doi: 10.1021/acs.chemmater.3c02740 (PMC10809410; doi:10.1021/acs.chemmater.3c02740)
Supplement: Supplementary file 1 — cm3c02740_si_001.pdf [file cm3c02740_si_001.pdf]

Supporting Information

Coherence in polycrystalline thin films of twisted molecular crystals

Yongfan Yang[†]*, *Alexander G. Shtukenberg*[†]*, *Hengyu Zhou*[†], *Christian Ruzie*[‡], *Yves Henri Geerts*[‡,§], *Stephanie S. Lee*[†], *Bart Kahr*[†]

[†] Department of Chemistry and Molecular Design Institute, New York University, New York, NY 10003, USA

[‡] Laboratoire de Chimie des Polymères, Faculté des Sciences, Université Libre de Bruxelles (ULB), Boulevard du Triomphe, CP 206/01, Brussels 1050, Belgium

[§] International Solvay Institutes of Physics and Chemistry, Université Libre de Bruxelles (ULB), Boulevard du Triomphe, CP 231, Brussels 1050, Belgium

Corresponding authors: yy3000@nyu.edu (Y. Yang); as5243@nyu.edu (A. G. Shtukenberg)

Table of contents

Materials and Methods

Figure S1. Optical image of twisted DPP-C12 spherulites with band boundary curves drawn in MATLAB.

Figure S2. Schematics of fiber organization in twisted crystals with in-phase and random distributions.

Figure S3. Polarized light optical images of DPP-C12 spherulites grown at different temperatures.

Figure S4. Growth rate and pitch as a function of growth temperature for DPP-C12.

Figure S5. SEM and AFM images of twisted BDT crystals.

Figure S6. Illustration of the twisted fiber organization mechanism.

Figure S7. Branching rate and fiber size for twisted DPP-C12 crystals with varied pitch.

Figure S8. Correlation between fiber thickness and branching rate for coumarin.

Figure S9. Measured fiber thickness for twisted DPP-C8, BDT, coumarin, δ-mannitol, and resorcinol with varied pitch.

Figure S10. Distribution of fiber thickness and pitch for twisted DPP-C12.

Figure S11. Measured fiber length and comparison of σ_x/P from measurement and model calculation for δ-mannitol.

Figure S12. SEM images of twisted films for BDT and δ-mannitol showing the 'flower' texture.

Figure S13. Illustration of flower model simulation procedure for twisted DPP-C12 film.

Figure S14. SEM images of twisted DPP-C12 crystals showing $2\theta = 30°$.

Figure S15. Boundary variance vs. band number for DPP-C12 pattern.

Figure S16. Boundary variance vs. band number and pattern width for DPP-C12.

Figure S17. Boundary variance vs. band number, $2\theta=1°$ for DPP-C12 pattern.

Table S1. Crystal data and structure refinement parameters for DPP-C12 single crystal.

Figure S18. OFET setup and transfer curves for twisted DPP-C12 OFET with pitch P = 40 μm.

Figure S19. Transfer curves for twisted DPP-C12 OFET with pitch P = 60 μm.

Figure S20. Schematic diagram of DPP-C12 twisted fibers and molecular packing in crystal.

Figure S21. High-resolution X-ray diffraction patterns for DPP-C12 crystals grown at different temperatures.

Figure S22. 2D X-ray diffraction patterns of twisted DPP-C12 crystals with flat-on and edge-on orientations.

Figure S23. DFT calculated carrier mobilities for DPP-C12.

Figure S24. Schematics of a resistor framework.

Table S2. DFT calculated transfer integral, hopping rate and mobility for DPP-C12.

Figure S25. Simulated electrical potential distribution of an ideal film.

Table S3. Relative conductance for simulated DPP-C12 patterns vs. boundary variance.

Figure S26. Relative conductance for simulated DPP-C12 patterns with different area of flat-on orientation.

Figure S27. Effect of the area of flat-on orientation on the relative conductance for simulated DPP-C12 patterns.

Figure S28. Electrical potential distribution of simulated DPP-C12 patterns vs. σ_x/P.

Figure S29. Dependence of relative conductance on the coefficient α and β.

Figure S30. Electrical potential distribution of optical images of DPP-C12 grown on OFET with P = 40 μm.

Figure S31. Electrical potential distribution of optical images of DPP-C12 grown on OFET with P = 60 μm.

Video S1. Growth of twisted DPP-C12 crystals from the melt.

Crystallographic Information File:

CCDC code: 2267758

Materials and Methods

Materials. Tetraphenyl lead (TCI America, 95%, melting point T_m = 230 °C), δ-mannitol (Sigma-Aldrich, 98%, T_m = 165 °C), coumarin (Sigma-Aldrich, 99%, T_m = 75 °C), resorcinol (Sigma-Aldrich, 99%, T_m = 110 °C), DPP-C8 (SunaTech Inc.; 95%, T_m = 145 °C) and DPP-C12 (SunaTech Inc.; 98%, T_m = 125 °C), were used without further purification. BDT (T_m = 75 °C) and additional sample of DPP-C12 synthesized by Christian Ruzie were provided by Prof. Yves Henri Geerts.

Powder X-ray Diffraction. X-ray microdiffraction (μ-XRD) was performed using a Bruker D8 Discover General Area Detector Diffraction System (GADDS) equipped with a VÅNTEC-2000 two-dimensional (2D) detector and a sealed Cu-Kα source (λ = 1.54178 Å). The X-ray beam was monochromated with a graphite crystal and collimated with a 0.5 mm capillary collimator (MONOCAP). The sample was loaded on a silicon chip for data acquisition and the sample-to-detector distance was 145 mm.

Polarized Absorption Spectra. Optical absorption by the films was measured using a CRAIC Technologies 508 PV microscope spectrophotometer in the range of 300-1000 nm equipped with a polarizer. Aperture sizes were chosen for tightly and loosely twisted crystals to maintain the ratio of aperture size to pitch. Crystallites orientations vary within the aperture by ca. 10°.

Scanning Electron Microscopy (SEM). Samples were mounted on conductive carbon tape, fastened to aluminum holders, and coated with 5 nm of gold. The images were recorded with a Carl Zeiss MERLIN field emission scanning electron microscope using standard detectors: Everhart-Thornley type and annular secondary electron with an acceleration voltage of 5 kV and current 110 pA.

Numerical simulations of fiber organization in banded spherulites. The spherulite was assumed to be formed by extreme fiber orientations, flat-on with a high branching rate, and edge-on with a low branching rate. Edge-on fibers change orientations to become flat-on randomly according to a Gaussian distribution with an average edge-on segment length equal to $P/2$ (P is pitch) and dispersion σ_P. The length of the ith fiber (i = 1, 2,…, M – total number of fibers used in simulation, M ~ 500) with an edge-on orientation calculated as half of the pitch $l_i = P_i/2 = bh_i/2$ (b = 543 in Figure 3d) assuming that thickness h_i is distributed normally with the mean thickness $\bar{h}$ and standard deviation σ_h. Edge-on fibers are usually loosely packed, the distances between them d_i were calculated based on the exponential distribution with the average fiber separation obtained from SEM images.

Fibers with flat-on orientations have a higher branching rate resulting in compact sector-like shapes ("petals"), with a radius $P/2$ and a constant opening angle 2θ. For DPP-C12, this angle was directly measured from SEM images as $2\theta \approx 30°$ (Figure S15). This angle is similar to the value calculated from average misorientation angle γ = 1.27° and the geometric relationship $2\theta = \mathrm{asin}(\frac{P}{h}(\gamma)^2) = 25°$.[1] Once a new petal advances at half the pitch $P/2$, the flat-on fibers become edge-on and the cycle starts anew.

Neighboring petals typically collide so that the resulting pattern is determined by a geometrical selection process. The simulations were performed as follows:

Step I: Generation of the edge-on orientations. Flower petal texture refers to the flat-on orientation with high branching rate and its stem position is the ending point of the edge-on orientated fiber. Figure S13a illustrates the distribution of edge-on oriented crystal fibers, where the thickness h_i follows the normal distribution determined by the mean h and standard deviation σ_h. The fiber length is assigned as a half of the pitch P_i, which is a function of thickness h_i ($P = bh$) – excepting the transition region between edge-on and flat-on orientations. The distance between edge-on fibers d_i follows an exponential distribution with a mean distance between fibers defined by the average mean h: $d = 20h$. All-possible flower stems generating points are shown in Figure S13b.

Step II: Two rays with the opening angle $2\theta = 30°$ are generated from each of the generating points to give the flower shape (Figure S13c). This opening angle was measured from SEM images.

Step III: When two flowers collide, they are superimposed on top of each other. The radius for all flowers is a half of the average pitch $P/2$. The generating points are taken as the lowest points so that the generated flower area can occupy the pattern width. The flowers cannot be generated at higher initial points because the corresponding space will be already filled by flowers generated from the lower initial points. Therefore, the first band is simulated and the top arcs represent the band boundary (Figure S13d).

Step IV: Figure S13e displays the second band where the distance Δx_i between the initial position of the second generation of flowers and the arc of the first ones is half of pitch P_i, which is a function of thickness h_i (Same as Step I).

Step V: The following bands were generated using the same protocol but with the final positions of the fibers in the previous cycle used as initial positions for the next cycle.

Mueller Matrix Microscopy. Crystal optical properties were established with a Mueller matrix microscope, described previously,[2,3,4,5] using a xenon arm lamp. Linear retardance |LR| is defined by eq. S1 and represents a phase differences between orthogonally plane polarized light (expressed in rad) and proportional to the path length, L.

$$\mathrm{LR} = 2\pi(n_{0°} - n_{90°})L/\lambda \tag{S1}$$

where $n_{0°}$ and $n_{90°}$ are the refractive indices for orthogonal polarizations, $\lambda = 550$ nm is wavelength of light.

Atomic Force Microscope (AFM). AFM measurements were performed in contact mode using a Bruker Multimode 8 AFM instrument equipped with Bruker DNP-10 Si_3N_4 tips on silicon nitride cantilevers with a spring constant of 0.12 N/m (triangular tip B, 205 μm length, 40 μm width). The images were analyzed by the software package Gwyddion.[6]

Linear Retardance simulation. The simulation of linear retardance used a model developed to compute the optical properties of twisted spherulites using MATLAB.[3,4,7] This model computed the Mueller matrix of materials based on their electric permittivity tensor and misorientation of all fibers along the light pathway. This algorithm calculated the optical properties of a stack of fibers along the *z*-direction normal

to the plane of a glass slide in the *xy*-plane, hence, band boundary incoherence along the *z*-direction was considered a factor to influence linear retardance. Boundary incoherence along *z*-direction (σ_z/P) was assumed to be the same as incoherence along *x*-direction (σ_x/P) obtained from optical images. The pitch P was obtained from optical images and the fiber thickness h was measured from SEM images. The thickness of the film (h_{total}) was measured by AFM, so that the number of fibers on the *z*-direction (#*h*) was calculated as h_{total}/h. Linear retardance (|LR|) of coumarin was measured by Mueller matrix microscope. The optical properties were measured and calculated at λ = 550 nm. With all aforementioned input parameters, the simulated band patterns from Figure 5e-h in the main text were used to generate the LR images. The Matlab program can be found on Github: https://github.com/hz2134-chem/KahrWardGroup/tree/main/Band_Coherence_Optics.

Organic Field Effect Transistor (OFET) Fabrication and Electrical Characterization. For OFETs, a bottom-gate, bottom-contact structure was used, where the bottom gate consisted of highly-doped silicon with 300 nm of thermally grown SiO_2 as the gate dielectric (Figure S18a). Source and drain contacts were patterned by photolithography and deposited by ε-beam evaporation (5 nm Cr followed by 50 nm Au) and a channel length of 100 μm. The channel length of 100 μm and width of 500 μm was chosen in order to have much higher channel resistance compared than contact resistance so as to minimize the influence of contact resistance on the measured mobility values.[8,9] Prior to vacuum deposition, the substrates were cleaned in an ultrasonic bath for 15 min in DI water, acetone and isopropyl alcohol. The twisted crystals were grown from the melt on the devices and were measured by Keithley 2636B system source meter. OFET measurements were performed at room temperature. Previous research proved that oxidation does not affect measurements on samples prepared under ambient conditions.[14,10] Hole mobility μ measurements were taken in the saturation regime when drain-source voltage V_D = -80 V, following $I_D = \frac{WC_i}{2L}\mu(V_G - V_T)^2$ expression.[11] Here, the channel width W = 500 μm, length L = 100 μm and gate dielectric capacitance $C_i = 1.06 \times 10^{-8}\,\text{F/cm}^2$, V_G is gate voltage varying from 0 to -80 V. Crystallization on electrodes proceeded in the same way as on glass slides.

Crystal Orientation Determination of DPP-C12. The DPP-C12 crystal structure (Refcode RAJLIB[12]) was published earlier. Herein, we redetermined its crystal structure by single crystal X-ray diffraction (SCXRD) analysis in the monoclinic space group $P2_1/c$ (Supplementary Table 1). The aromatic groups are stacked along the *b*-axis with the distance between the mean π–planes of 3.53 Å (Supplementary Figure 20). 2D XRD patterns with a focused beam indicated that light red bands observed between cross polarizers correspond to flat-on (100) orientations and dark bands correspond to an edge-on (001) orientations. The growth direction is <010> that corresponds to the intermolecular π-π stacking *b* axis (Supplementary Figs. 21, 22).

Density Functional Theory (DFT) Calculations. DPP-C12 molecules crystallize into a layered herringbone packing motif, which provides 2D transport only within the basal stacked organic layers, while transport between layers is blocked by the -C_{12} alkyl chains, similarly to many π-conjugated molecules.[13,14] In this case, the carrier mobility can be calculated from eq. S5[15]

$$\mu_\phi = \frac{e}{2k_BT}\sum_i r_i{}^2 W_i P_i \cos^2\gamma_i \cos^2(\theta_i - \phi) \tag{S5}$$

where e and k_B are the electron charge and Boltzmann constant, respectively, and temperature T = 300 K. Index i represents a specific hopping path with hopping distance r_i (centroid-to-centroid). γ_i is the angle between the hopping path i and b-c plane, herein 0°. θ_i is the angle of the hopping path i projected on b-c layer relative to the reference b axis, ϕ is the orientation angle of transistor channel to the reference b axis. $P_i = W_i / \sum_i W_i$ is the hopping probability. The hopping rate W_i is calculated from Marcus-Hush theory[16,17] as eq. (S6)

$$W_i = \frac{t_i{}^2}{h}\left(\frac{\pi}{\lambda k_BT}\right)^{\frac{1}{2}}\exp\left(-\frac{\lambda}{4k_BT}\right) \tag{S6}$$

where h is the Planck constant, t_i is the transfer integral along the hopping path i, and λ is the reorganization energy.

The transfer integrals t_i along the hopping path i are estimated with an energy-splitting approach[18] by considering the orbital energies, E, of the corresponding dimer,

$$t_{12}{}^{\mathrm{h}} = \frac{E_{\mathrm{HOMO}} - E_{\mathrm{HOMO-1}}}{2} \tag{S7}$$

$$t_{12}{}^{\mathrm{e}} = \frac{E_{\mathrm{LUMO+1}} - E_{\mathrm{LUMO}}}{2} \tag{S8}$$

Reorganization energy λ for hole and electron transfer was calculated using the four-point rule[11,19,20]

$$\lambda = \lambda_1 + \lambda_2 = (E_+^* - E_+) + (E_0^* - E_0) \tag{S9}$$

where E_0 is the energy of the neutral molecule in the lowest energy geometry, E_0^* is the energy of the neutral molecule with the charged geometry, E_+ is the energy of the cation molecule in the lowest energy geometries, E_+^* is the energy of the cation molecule with the neutral geometry.

Transfer integrals t_i, hopping rates W_i, and mobility μ_i along each hopping path i are summarized in Table S2. The calculations were implemented using the DFT with the B3LYP functional and the 6-31G(d) basis set, in the Gaussian 16 package.[21]

Effective Conductivity Calculation of a Resistor Network. The network is a rectangular region, consisting of A (row) × B (column) nodes and a series of resistors oriented horizontally and vertically between two adjacent nodes (Figure S24). The goal is to calculate the conductivity from the left side to the right side with an electrical potential applied from left to right. Based on Kirchhoff's and Ohm's laws, the total current flowing in and out of each node i is

$$I_i = \sum_i C_{ij}\left(U_i - U_j\right) = 0 \tag{S10}$$

where C_{ij} is the conductance of the resistor connecting node i and surrounding node j, and U_i and U_j are voltages at nodes i and j, respectively. The minimum value of electric power dissipated, P_{opt}, can be found from minimization of the quadratic form

$$P_{\mathrm{opt}} = C_{\mathrm{tot}}(U_{\mathrm{L}} - U_{\mathrm{R}})^2 = \min \sum_{\mathrm{resistor}} C_{ij}(U_i - U_j)^2 \tag{S11}$$

Here, C_{tot} is the total conductance of the network from the left to the right side, and U_{L} and U_{R} are the applied electrical voltages at the left and the right sides, respectively.

When applying $U_{\mathrm{L}} = 1$ V and $U_{\mathrm{R}} = 0$, the total conductance can be expressed as

$$C_{\mathrm{tot}} = \min \sum_{\mathrm{resistor}\ ij} C_{ij}(U_i - U_j)^2 \tag{S12}$$

Eq. (S12) can be also rewritten as

$$C_{\mathrm{tot}} = \min < \vec{U}, M^{\mathrm{T}} D M \vec{U} > \tag{S13}$$

where M is a matrix

$$M(r,y) = \begin{cases} 1 & \text{for } \ y = i \\ -1 & \text{for } \ y = j \\ 0 & \text{for } \ y \neq i \text{ or } j, \end{cases}$$

(S14)

in which the number of columns is equal to the number of nodes A × B and the number of rows is the number of resistors 2A×B-3A-B+2. For a resistor r connecting two nodes i and j, D is a diagonal matrix whose dimension is equal to the number of resistors and diagonal elements associating to the conductance of the resistor r^{th} from node i to j, i.e. $D(r,r) = C_{ij}$. The pattern with only flat-on oriented fibers can be regarded as an ideal single crystal, because of strong branching and compact structure. Therefore, the conductance of horizontal and vertical resistors in the flat-on region was defined based on DFT calculated mobilities tensors. The conductance is a product of carrier mobility μ and carrier concentration c. The carrier concentration is not known but was assumed to be the same in all DPP-C12 films.

For flat-on oriented fibers,

$$D(r,r) = \begin{cases} \mu_{22} & \text{for the horizontal resistor } r \\ \beta\mu_{33} & \text{for the vertical resistor } r \end{cases} \tag{S15}$$

where μ_{22} and μ_{33} are the elements of mobility tensors at positions (2, 2) and (3, 3) in Table S2, respectively. β is a parameter to describe the effect of boundaries between fibers when the electric field is applied perpendicular to the fiber elongation direction.

For edge-on orientated fibers,

$$D(r,r) = \begin{cases} \alpha\mu_{22} & \text{for the horizontal resistor } r \\ \mu_{11} = 0 & \text{for the vertical resistor } r \end{cases} \tag{S16}$$

where $0 < \alpha < 1$ is the parameter to describe weaker charge transport along fiber elongation direction for edge-on orientated fibers compared to flat-on orientated crystals. $\alpha < 1$ because loosely compacted fibers with edge-on orientations are separated by wider gaps and can block the charge transport to a greater extent.

The size of simulated DPP-C12 patterns from *Model of Fiber Organization* in section 2.4 with the same pitch P but different σ_x/P was 100 μm in length and 500 μm in width, comparable to the channel size of organic field effect transistor (OFET) measurement (see the following section). The pattern images with the resolution of 800 × 4000 pixels were converted to binary images composed of elements 0 and 1 using a luminance threshold of 0.35. Element 1 was defined as white for edge-on orientations, and element 0 was defined as red for flat-on orientation. Each binary image was divided into 100 × 500 subsections, and each subsection determined the conductance of two resistors on top and right as marked red in Figure S24.

Applying matrixes M and D into eq. (S5), yields the electric potential U_i at each node i and the total conductance C_{tot} of the films from the left to the right side was determined. The calculations were performed in MATLAB using the `Quadprog` optimization routine.

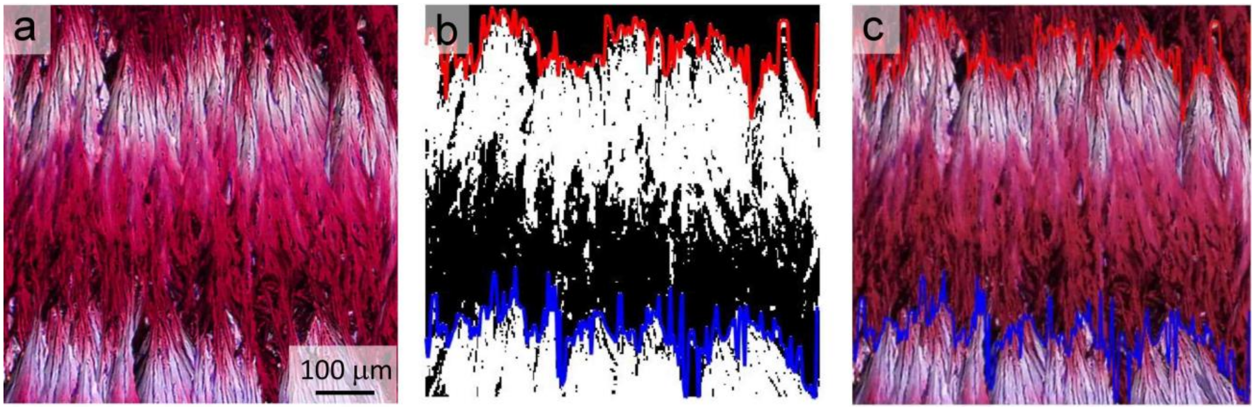

Figure S1. (a) Raw optical image between crossed polarizers, (b) binary black and white image with boundary curves in blue and red and (c) optical image with the band boundary curves from (b) are transferred to the raw image (a) for twisted DPP-C12 crystals with P = 425 μm.

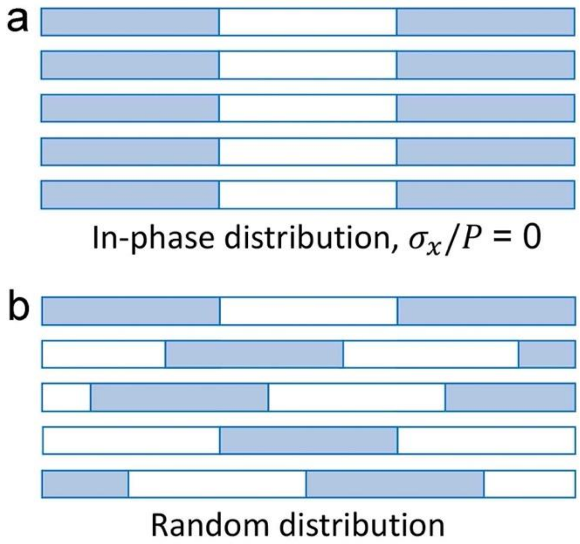

Figure S2. Schematics of fiber organization in twisted crystals following (a) in-phase distribution and (b) uniform random distribution.

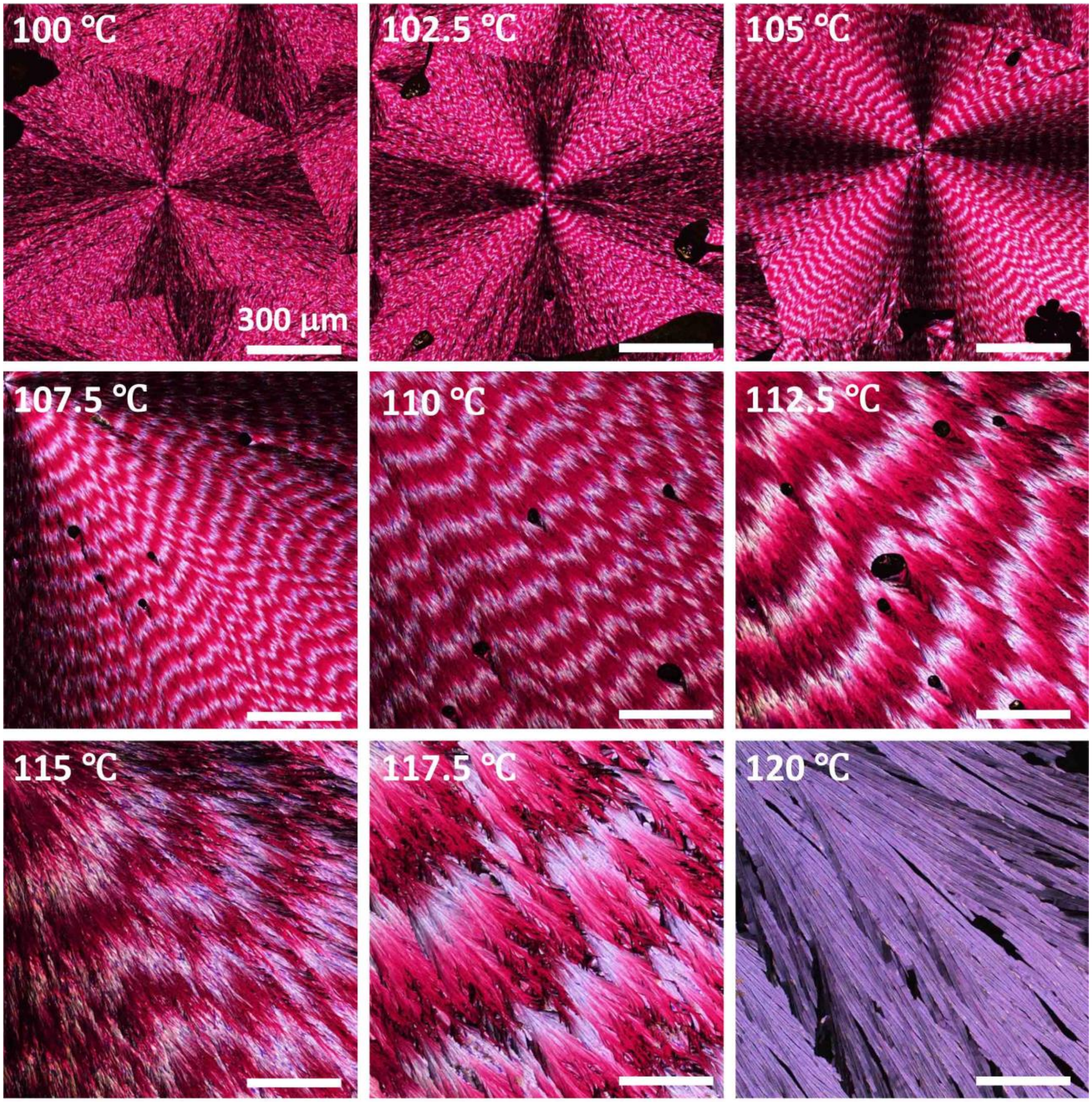

Figure S3. Optical images of DPP-C12 spherulites between crossed polarizers grown at temperatures indicated. Scale bar = 300 μm.

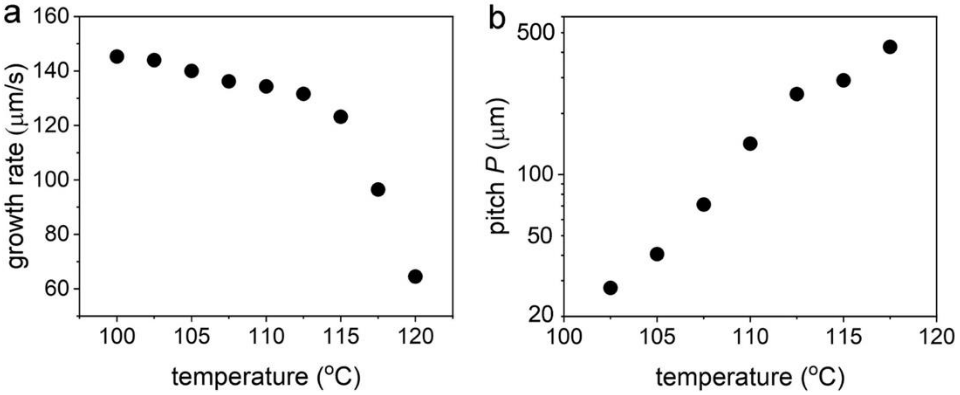

Figure S4. (a) Growth rate and (b) pitch P as a function of growth temperature for DPP-C12.

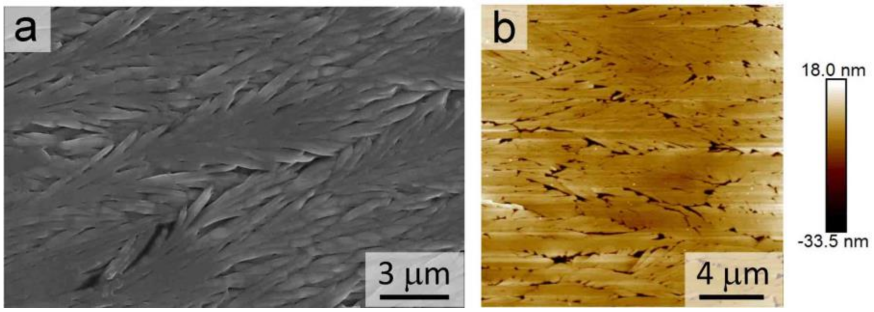

Figure S5. (a) SEM image of banded BDT spherulite with P = 80 μm. (b) AFM image of banded BDT spherulite with P = 20 μm. This simultaneous bending and twisting of crystallites[14] are likely responsible to very high boundary incoherence.

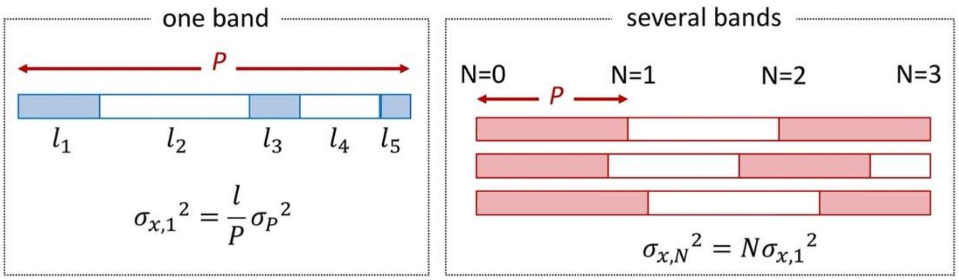

Figure S6. Illustration of fiber organization in twisted crystals for the first band and for more bands.

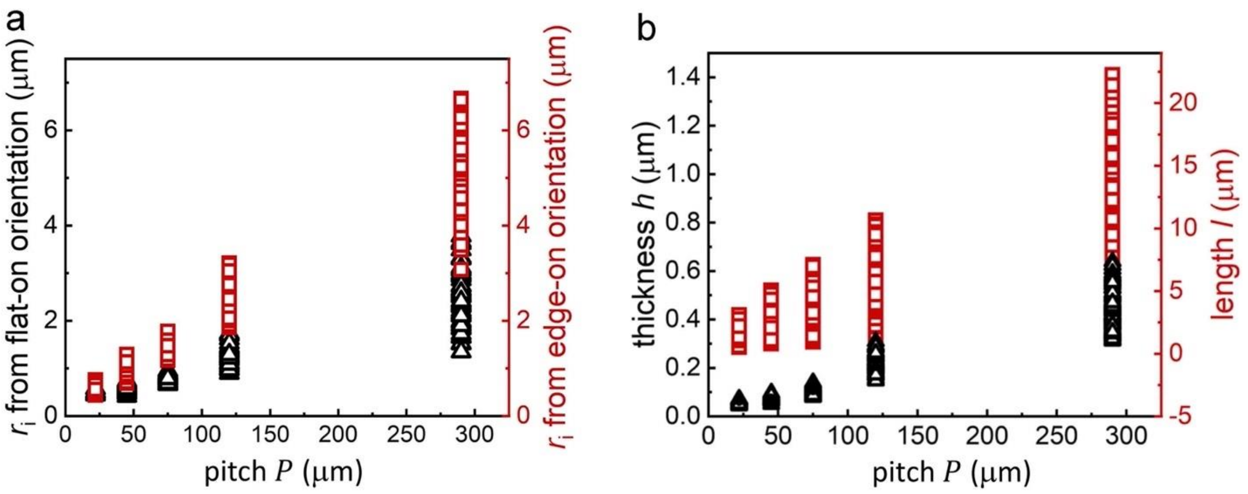

Figure S7. (a) Distance between two successive branching events r_b from edge-on and flat-on fibers and (b) fiber thickness h and length l in DPP-C12 spherulites measured from SEM images.

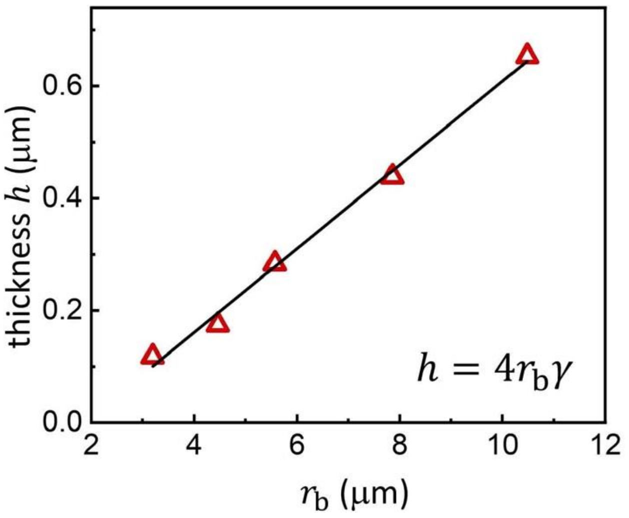

Figure S8. Fiber thickness h versus distance between two successive branching events r_b determined from AFM images for coumarin spherulites. γ is the average misorientation angle.

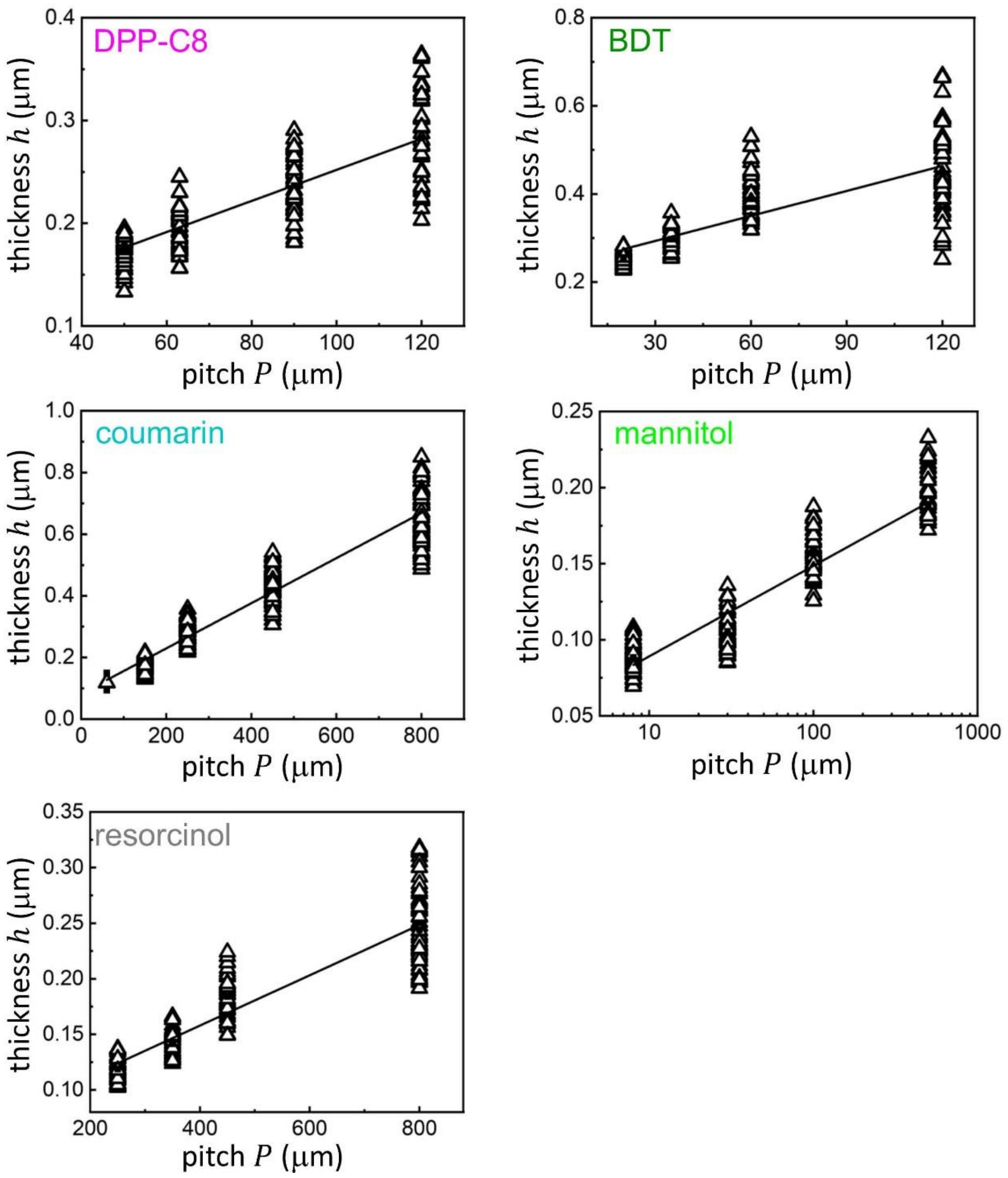

Figure S9. Experimentally measured fiber thickness h of twisted crystals with varied pitches P from SEM images for DPP-C8, BDT, coumarin, δ-mannitol and resorcinol.

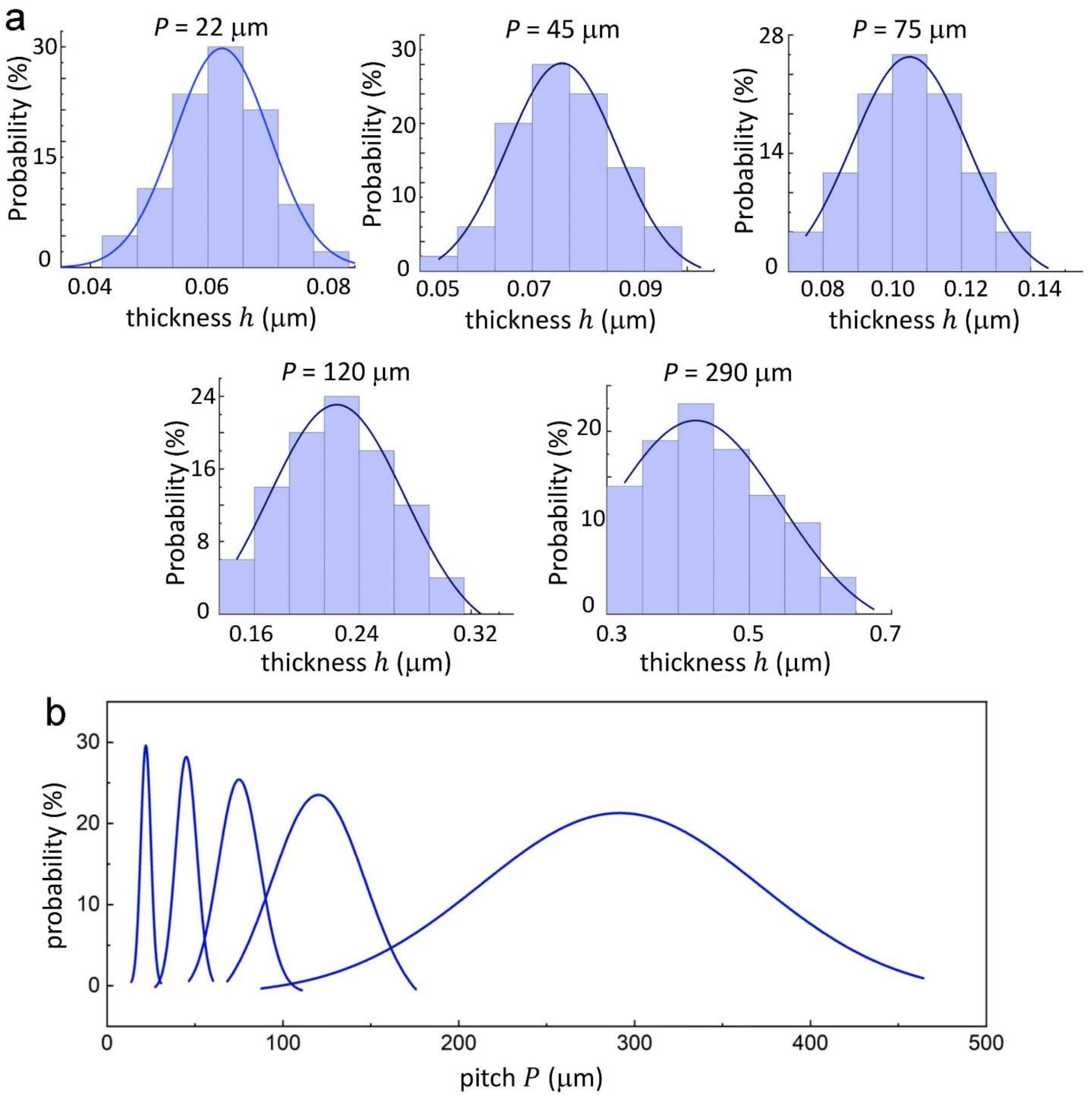

Figure S10. (a) Distribution of fiber thickness h for twisted DPP-C12 crystals with varied pitch P approximated by the Gaussian distribution and (b) pitch distribution calculated based on thickness distribution and relationship $\frac{\sigma_P}{\bar{P}} = \frac{\sigma_h}{\bar{h}}$.

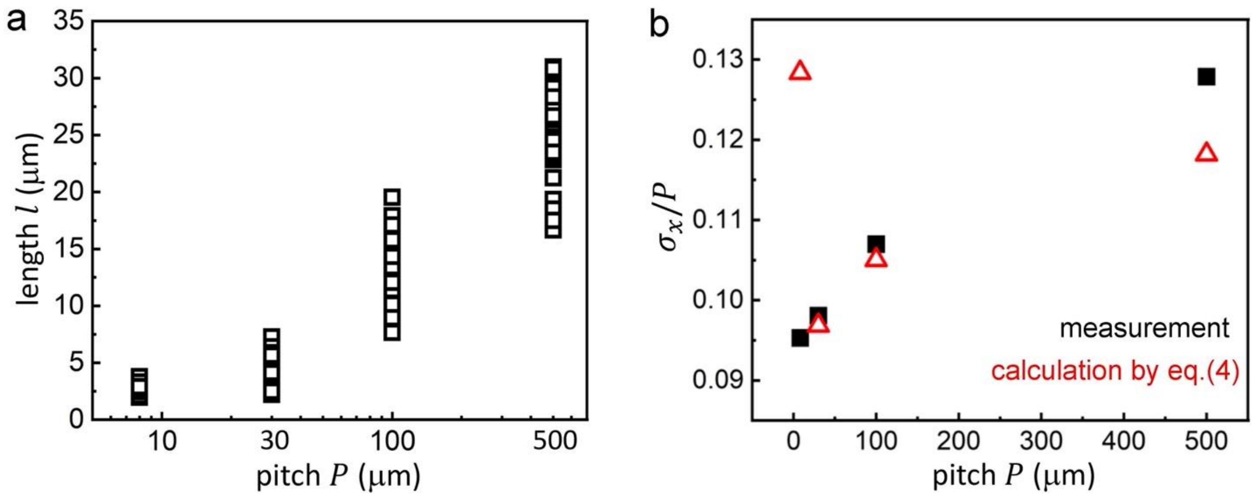

Figure S11. (a) Experimentally measured fiber length l for δ-mannitol crystal with varied pitches from SEM images. (b) Experimentally determined (black square) versus predictions from eq. (4) (red triangle) in the main text. For σ_x/P twisted crystals with larger pitches, there are a lot of branching, while for smaller pitch, the branching is lower, leading to the larger fiber length and thus big variance between the measurement and calculation.

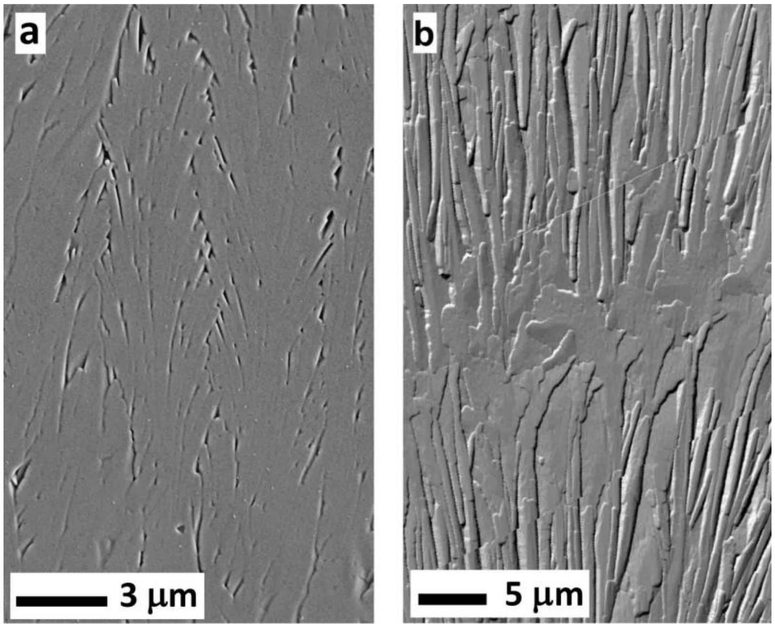

Figure S12. SEM images of twisted films for (a) BDT[14] and (b) δ-mannitol[22] showing the flower texture. The image (b) is reproduced with the permission from ref. 22. Copyright 2012, American Chemical Society.

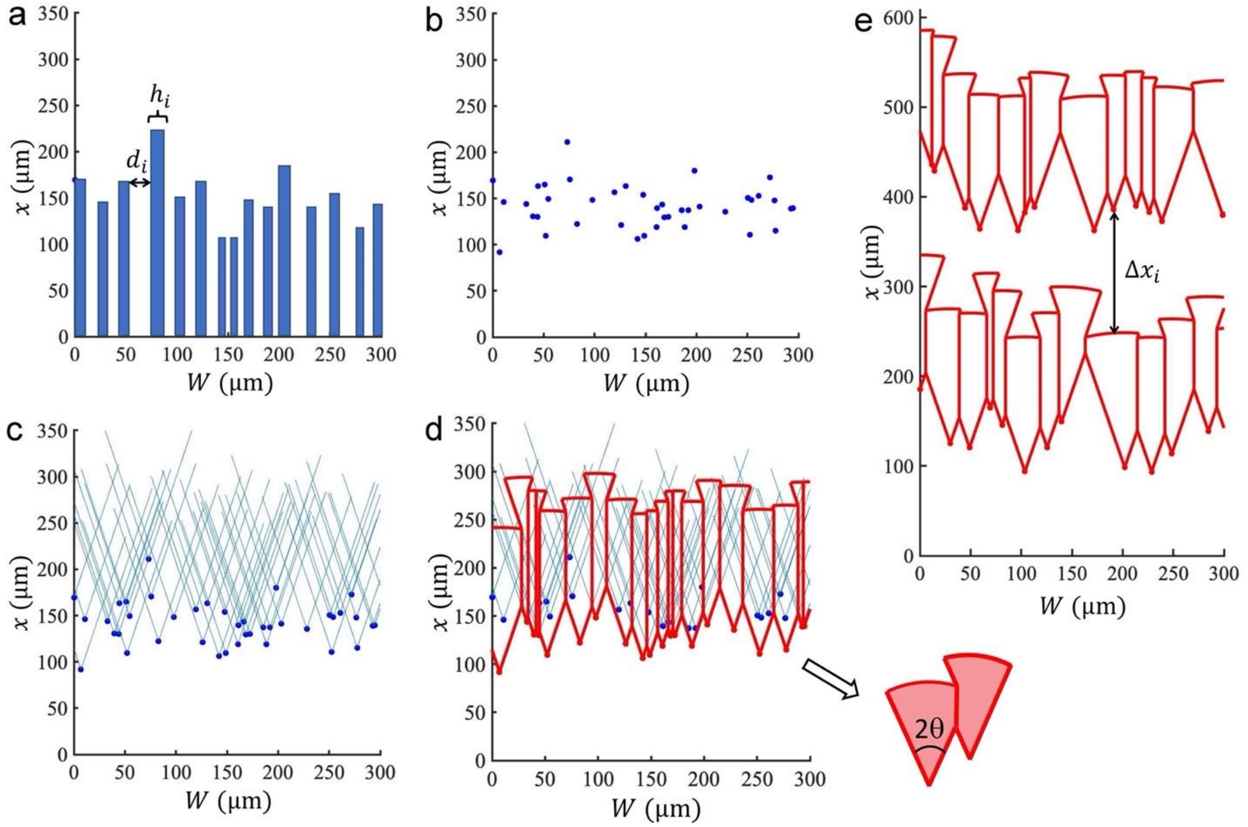

Figure S13. Illustration of 'flower model simulation' procedure for twisted DPP-C12 film (P = 300 μm). (a) Schematics of fiber organization with edge-on orientation and (b) all-possible generating points of flower stems (Step I). (c) Rays from each initial points with $2\theta = 30°$ (Step II). (d) Selection of initial points and the generated flower texture for the first band (Step III). The right bottom schematics illustrates the growth of neighboring flowers when they collide. (e) The simulated flower textures for film with two bands (Step IV).

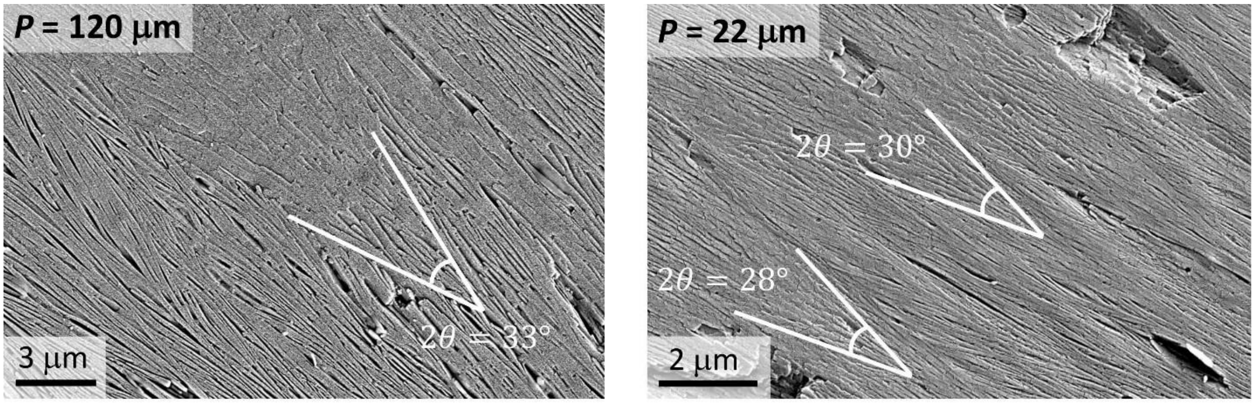

Figure S14. SEM images of twisted DPP-C12 crystals showing the opening angles 2θ for both large pitch P = 120 μm and small pitch P = 22 μm.

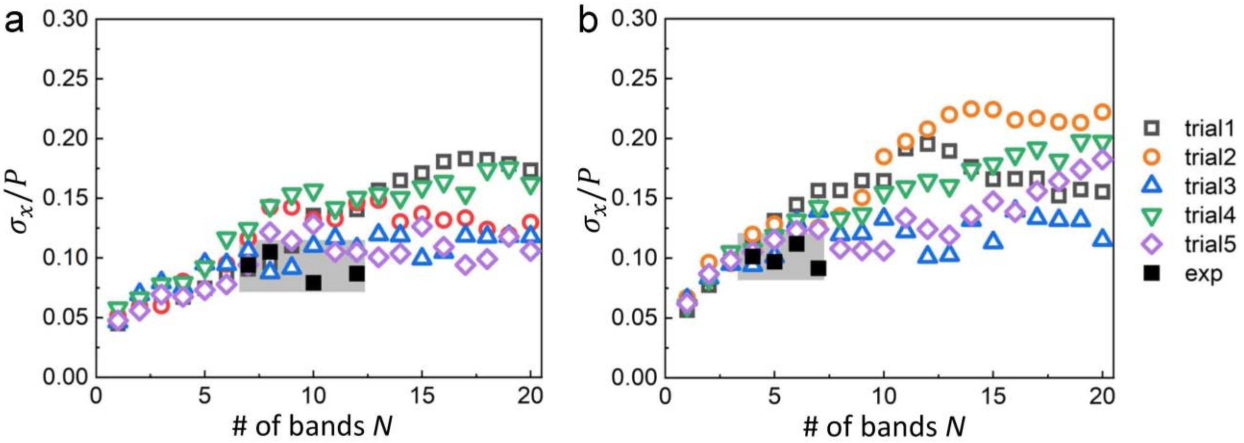

Figure S15. Simulated boundary incoherence σ_x/P (open symbols) as a function of the band number N for DPP-C12 pattern width W = 2P and (a) P = 75 μm and (b) P = 120 μm. Black solid squares are experimental data.

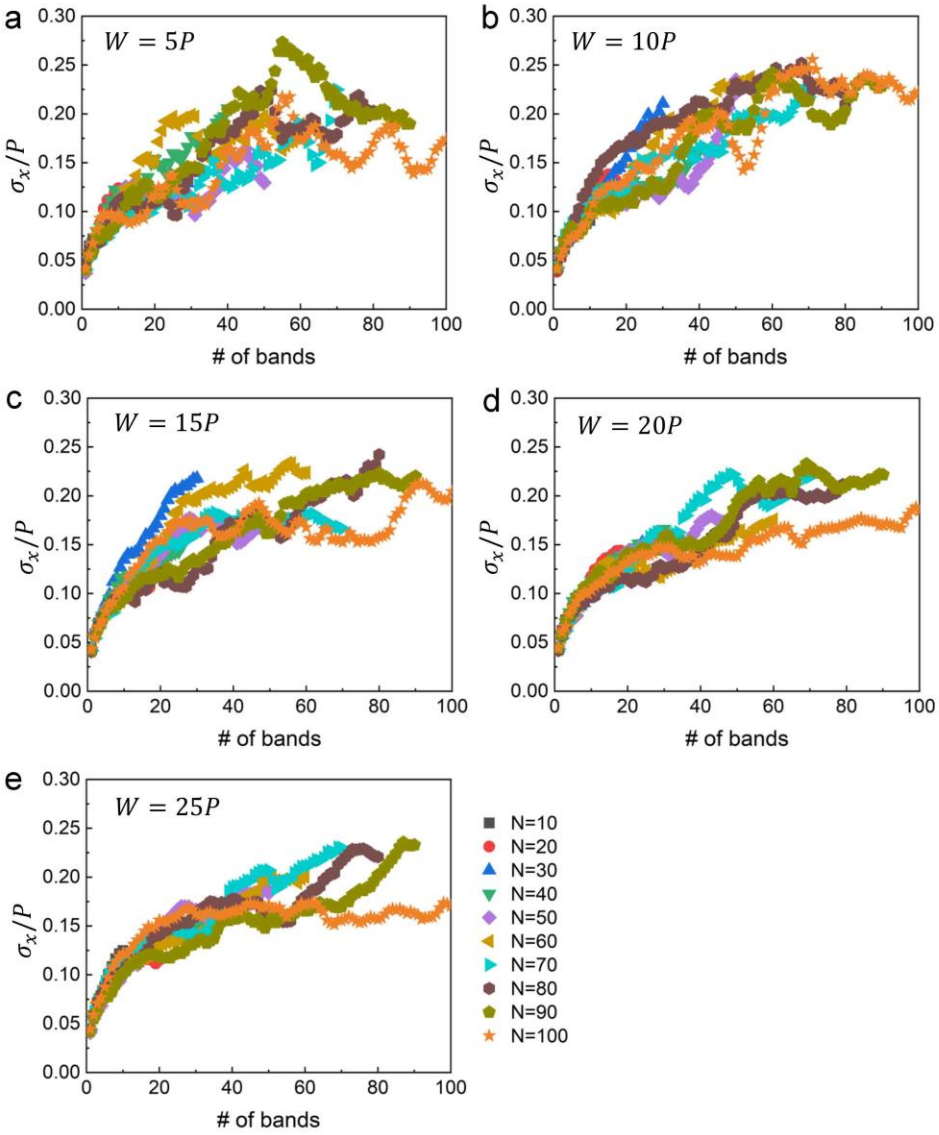

Figure S16. Simulated boundary incoherence σ_x/P as a function of band number N and pattern width (a) $W = 5P$, (b) $W = 10P$, (c) $W = 15P$, (d) $W = 20P$, (e) $W = 25P$. $P = 45$ μm and $2\theta = 30°$. Different colours correspond to different simulation trails.

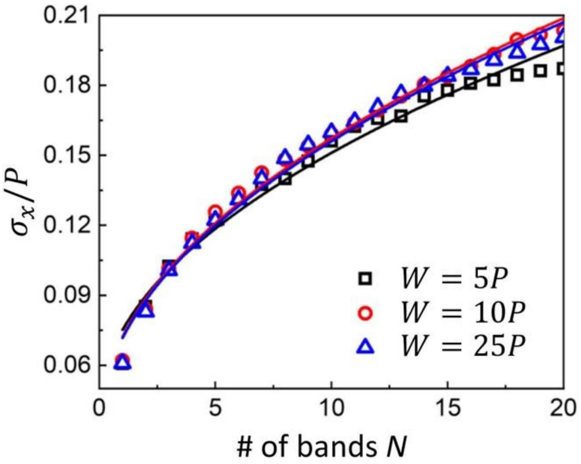

Figure S17. Simulated boundary incoherence σ_x/P as a function of band number N for $2\theta = 1°$ (dots) and their fit to $\sigma_x/P \propto N^{0.5}$ for different pattern widths W; $P = 45$ μm.

Table S1. Crystal data and structure refinement parameters for DPP-C12 single crystal.

Material	DPP-C12
Formula	$C_{38}H_{56}N_2O_2S_2$
Formula weight	636.97
Temperature / K	100
Crystal system	Monoclinic
Space group	$P2_1/c$
a / Å	21.431(8)
b / Å	5.099(2)
c / Å	15.874(6)
α / °	90
β / °	94.361(1)
γ / °	90
V / Å^3	1729.5
Z	2
D_c / g·cm^{-3}	1.223
μ / mm^{-1}	2.745
F (000)	692
Unique reflections	3065
R_1[a]	0.1509
wR_2[b] (all data)	0.2604
GOF	1.422

[a] $R_1 = \sum\left||F_0| - |F_c|\right| / \sum |F_0|$; [b] $wR_2 = \left\{\sum[w(F_0{}^2 - F_c{}^2)^2] / \sum[w(F_0{}^2)^2]\right\}^{1/2}$

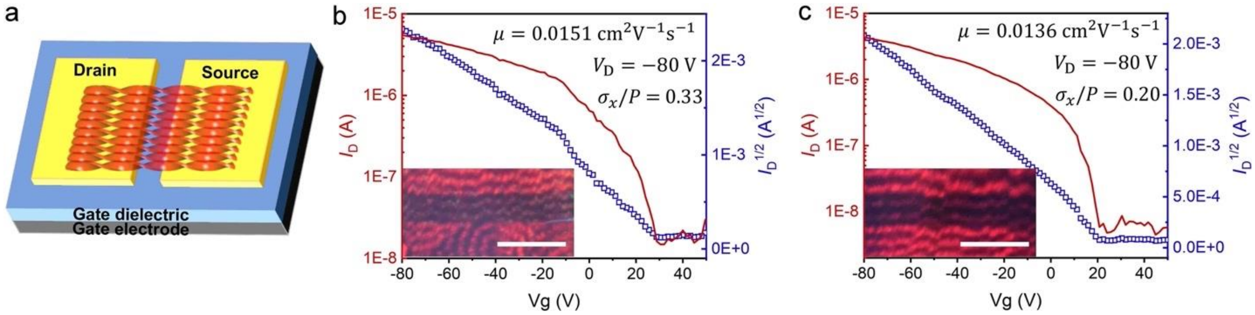

Figure S18. (a) Schematics of bottom-gate bottom-contact OFET with twisted DPP-C12 crystals with P = 40 μm grown along current flow direction from the drain to the source. Transfer curves in (b) boundary incoherence σ_x/P = 0.33 and hole mobility $\mu = 0.0151\ \mathrm{cm^2V^{-1}s^{-1}}$. Transfer curves in (c) boundary incoherence σ_x/P = 0.20 and hole mobility $\mu = 0.0136\ \mathrm{cm^2V^{-1}s^{-1}}$. The scale bar is 200 μm. Gate dielectric capacitance per unit area is $C_i = 1.06 \times 10^{-8}\ \mathrm{F \cdot cm^{-2}}$. The more coherent film (c) has a hole mobility that is smaller by 10% of the less coherent film (b).

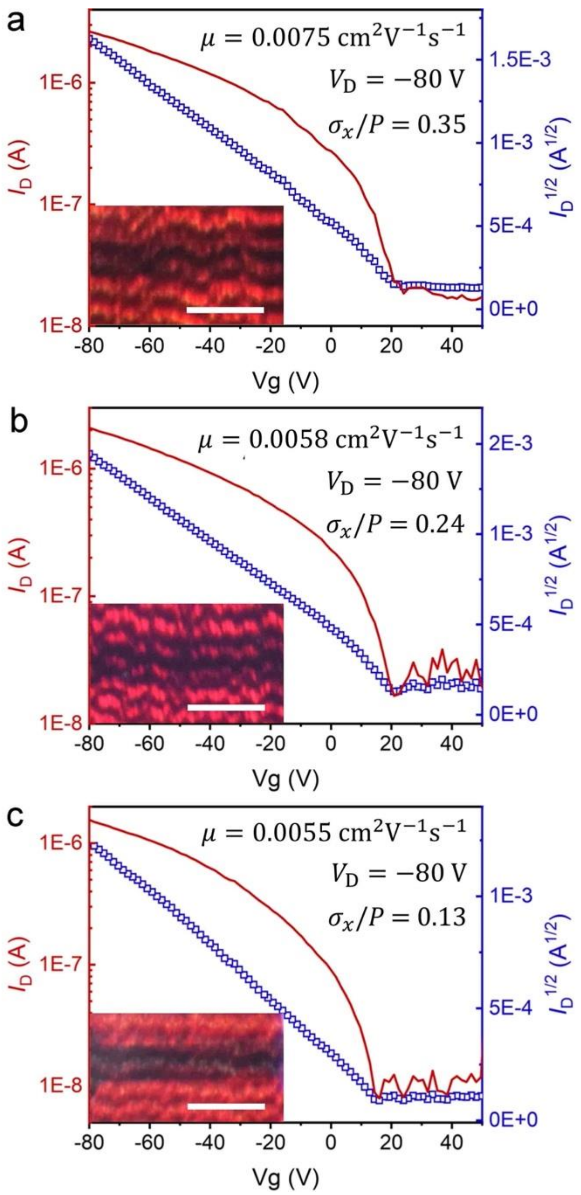

Figure S19. Transfer curves of twisted DPP-C12 OFET with pitch P = 60 μm. (a) Boundary variance σ_x/P = 0.35 and hole mobility $\mu = 0.0075\ \mathrm{cm^2V^{-1}s^{-1}}$. (b) Boundary variance σ_x/P = 0.24 and hole mobility $\mu = 0.0058\ \mathrm{cm^2V^{-1}s^{-1}}$. (c) Boundary variance σ_x/P = 0.13 and hole mobility $\mu = 0.0055\ \mathrm{cm^2V^{-1}s^{-1}}$. The scale bar is 200 μm. Gate dielectric capacitance per unit area $C_i = 1.06 \times 10^{-8}\ \mathrm{F \cdot cm^{-2}}$. Hole mobility increases with incoherence as in Figure S18.

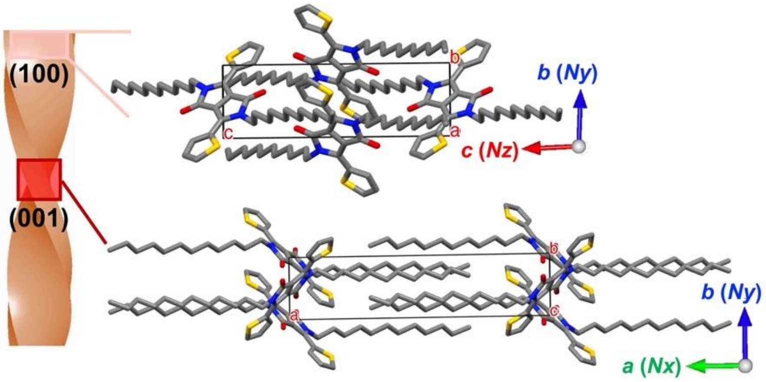

Figure S20. Schematic diagram of DPP-C12 twisted fibers along with molecular packing in the crystal structure.

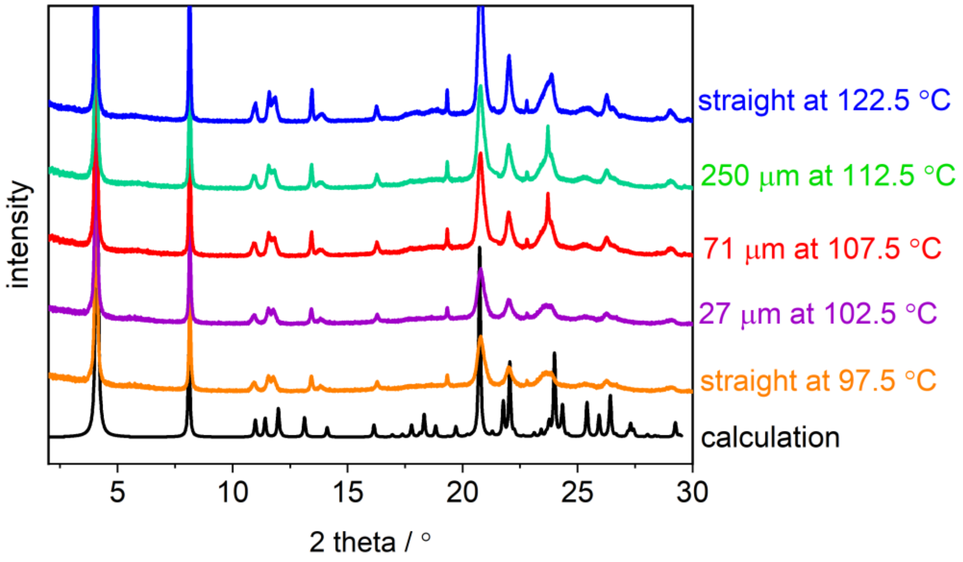

Figure S21. High-resolution X-ray diffraction patterns measured at room temperature of DPP-C12 crystals grown from the melt at different temperatures with varied pitch and the simulated patterns generated using the software Mercury (v3.10.1, 2018, Cambridge Crystallographic Database) based on single crystal structures (CCDC code: 2267758).

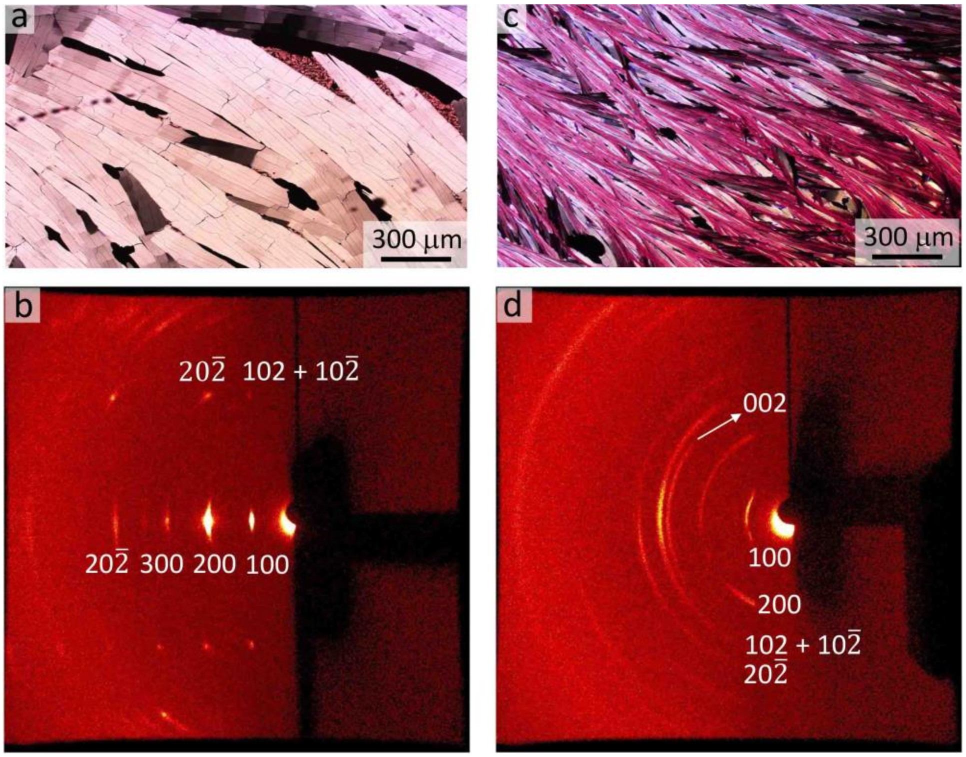

Figure S22. 2D X-ray diffraction patterns of DPP-C12 spherulites (P > 1000 μm) on the film with (a, c) flat-on orientation and (b, d) edge-on orientation collected with Bruker D8 Discover GADDS micro-diffractometer in reflection mode. The incident beam is in the plane containing the fiber elongation direction.

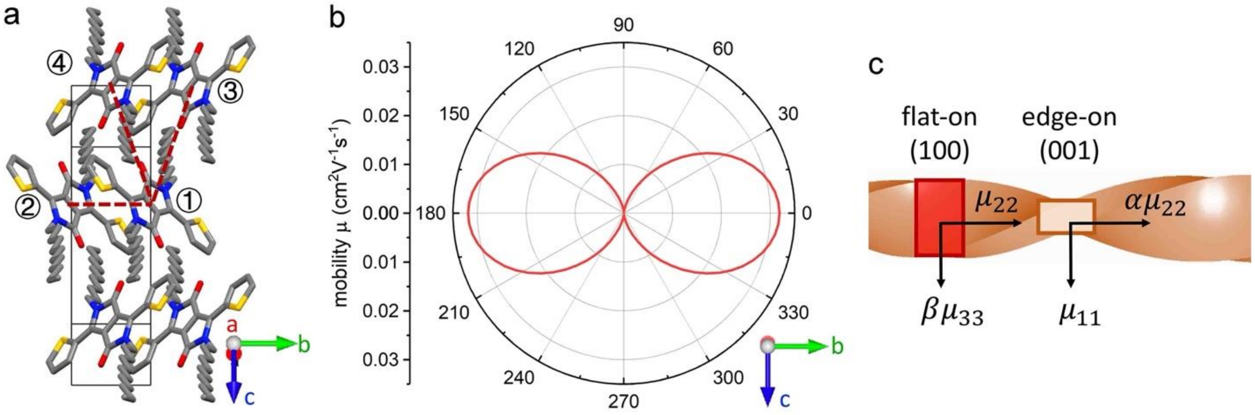

Figure S23. DFT calculation of carrier mobility for DPP-C12 crystals. (a) Crystal structure in the crystallographic basis {a, b, c} with three hopping paths 12, 13, and 14 shown by red dashed lines and corresponding molecules depicted by encircled numerals 1, 2, 3, and 4. (b) DFT calculated hole mobility tensor viewed along a*-axis. (c) Assigned conductance values parallel and perpendicular to the fiber elongation direction for flat-on (100) and edge-on (001) orientation for *Effective Conductivity Calculation.*

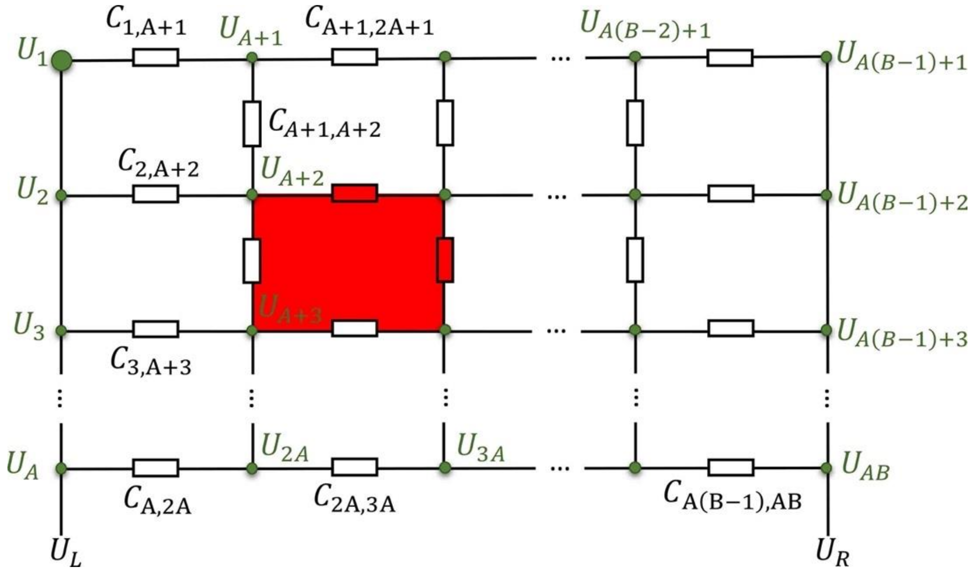

Figure S24. Resistor framework consisting of A × B nodes and a series of resistors orientated horizontally and vertically between two adjacent nodes. U_i is the electrical potential at each node and C_{ij} is the conductance of the resistor connecting node *i* and adjacent node *j*. The red subsection of the framework determines conductance of two resistors, the top and right, both are highlighted in red colour.

Table S2. DFT calculated transfer integral (t_i), hopping rate (W_i), mobility (μ_i), between DPP-C12 dimers along paths 12, 13 and 14 and mobility tensor in the orthogonal basis {$a*$, b, c} of a DPP-C12 crystal.

	Transfer integral t_i / meV	Hopping rate W_i / $\times 10^{11}$ s^{-1}	Mobility μ_i / cm^{2}V^{-1}s^{-1}
Path 12	56.20	46.1	0.032
Path 13	12.25	2.19	1.53×10^{-4}
Path 14	12.25	2.19	1.53×10^{-4}

Mobility tensors (μ_{ij}, cm^{2}V^{-1}s^{-1}) in the orthogonal basis {$a*$, b, c}					
μ_{11}	μ_{22}	μ_{33}	μ_{23}	μ_{13}	μ_{12}
0	0.032	2.79×10^{-4}	-1.83×10^{-6}	0	0

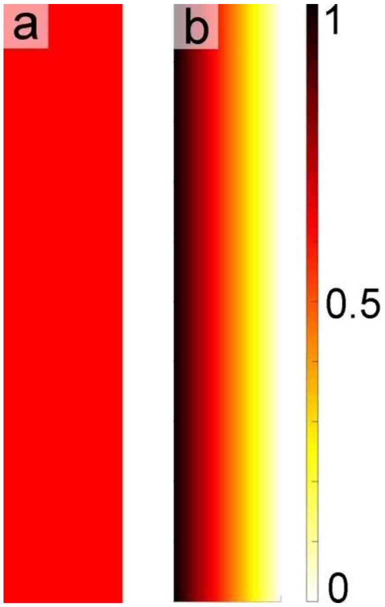

Figure S25. (a) Ideal film with only flat-on oriented fibers (red). (b) Simulated electrical potential distribution with applied electrical potential ΔU = 1 V from the left to the right side. α = 0.1 and β =1.

Table S3. Simulated relative conductance C/C_{ideal} for patterns vs. σ_x/P under the applied electric field ΔU = 1 V along fiber elongation direction <010>. α = 0.1 and β =1.

Pattern number	Relative conductance C/C_{ideal}				
	$\sigma_x/P = 0.078$	$\sigma_x/P = 0.111$	$\sigma_x/P = 0.138$	$\sigma_x/P = 0.187$	$\sigma_x/P = 0.270$
1	0.246	0.254	0.261	0.272	0.277
2	0.264	0.236	0.249	0.264	0.300
3	0.259	0.277	0.287	0.305	0.292
4	0.230	0.262	0.254	0.281	0.303
5	0.245	0.256	0.264	0.285	0.305
6	0.248	0.270	0.277	0.297	0.283
mean	0.249	0.259	0.265	0.284	0.295

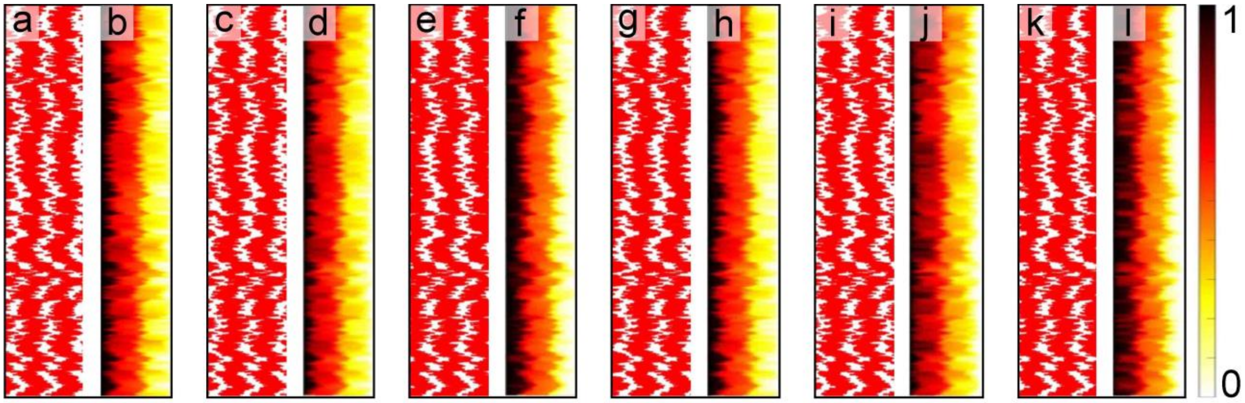

Figure S26. Effect of the relative area ratio of red region (flat-on fiber orientation) to the whole image S_{red}/S of on the relative conductance for simulated DPP-C12 patterns with σ_x/P= 0.187. (a, c, e, g, i, k) Simulated patterns starting from different position and (b, d, f, h, k, l) corresponding electrical potential distribution for hole transport under the applied electrical potential ΔU = 1 V from the left to the right side. (a) S_{red}/S = 0.644 and (b) C/C_{ideal} = 0.272. (c) S_{red}/S = 0.635 and (d) C/C_{ideal} = 0.264. (e) S_{red}/S = 0.705 and (f) C/C_{ideal} = 0.305. (g) S_{red}/S = 0.666 and (h) C/C_{ideal} = 0.281. (i) S_{red}/S = 0.639 and (j) C/C_{ideal} = 0.285. (k) S_{red}/S = 0.688 and (l) C/C_{ideal} = 0.297. α = 0.1 and β =1.

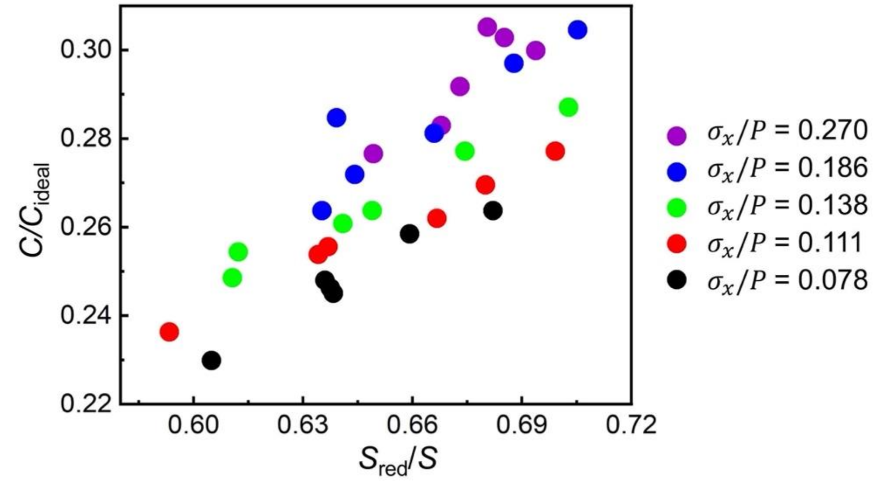

Figure S27. Dependence of relative conductance C/C_{ideal} on the area ratio of red region S_{red}/S for the simulated patterns with different boundary incoherence σ_x/P. α = 0.1 and β =1.

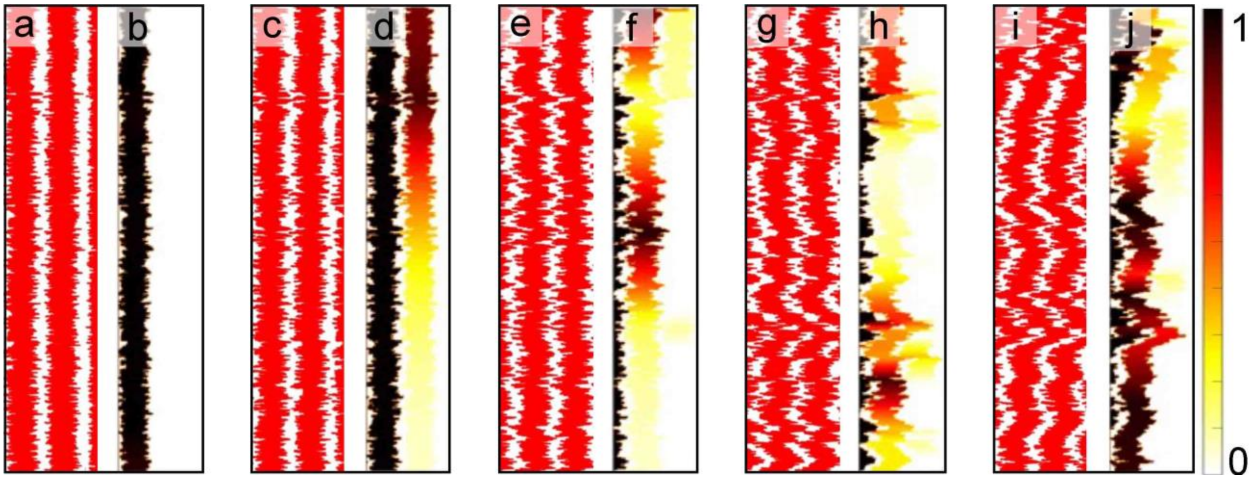

Figure S28. (a, c, e, g, i) Simulated patterns with varied σ_x/P and (b, d, f, h, j) calculated electrical potential distribution for hole transport under the applied electrical potential ΔU = 1 V from the left to the right side; α = 0 and β =1. (a) σ_x/P = 0.078 and (b) $C/C_{\mathrm{ideal}} = 1.73 \times 10^{-6}$. (c) σ_x/P = 0.111 and (d) $C/C_{\mathrm{ideal}} = 1.09 \times 10^{-4}$. (e) σ_x/P = 0.138 and (f) $C/C_{\mathrm{ideal}} = 2.09 \times 10^{-3}$. (g) σ_x/P = 0.187 and (h) $C/C_{\mathrm{ideal}} = 9.92 \times 10^{-2}$. (i) σ_x/P = 0.270 and (j) C/C_{ideal} = 1.17×10^{-1}.

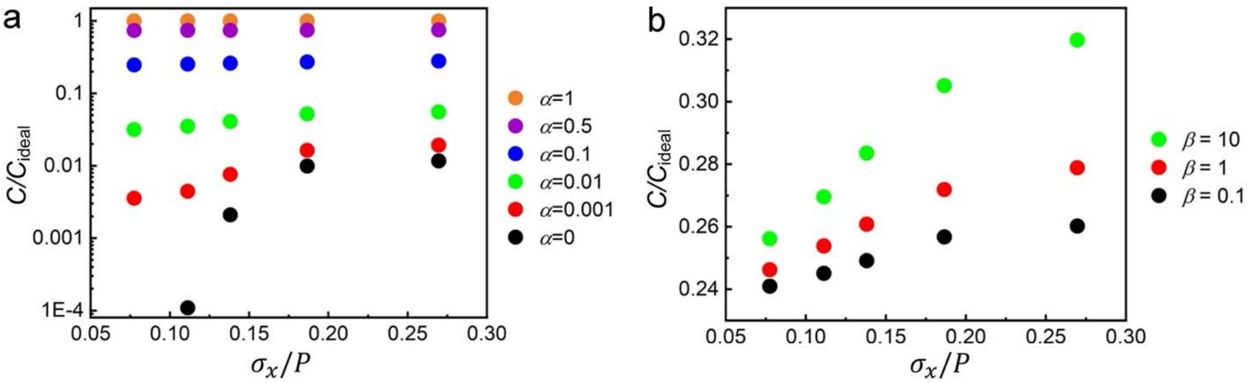

Figure S29. Dependence of relative conductance C/C_{ideal} for simulated patterns with different boundary incoherence σ_x/P on (a) varied coefficient α and constant β = 1 and (a) varied coefficient β and constant α = 0.1.

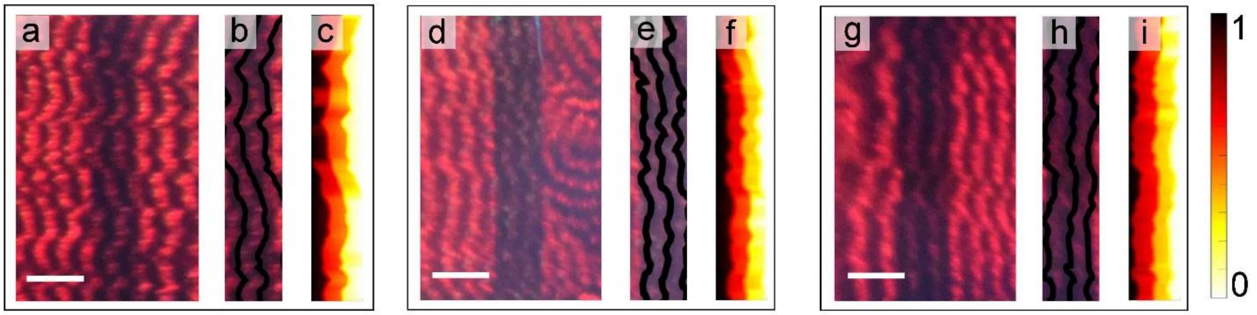

Figure S30. (a, d, g) Optical images of twisted crystals grown on OFET device. (b, e, h) Optical images combined with the boundary curves and (c, f, i) simulated electrical potential distributions. (b) σ_x/P = 0.42 and (c) C/C_{ideal} = 0.32. (e) σ_x/P = 0.33 and (f) C/C_{ideal} = 0.26. (h) σ_x/P = 0.20 and (i) C/C_{ideal} = 0.24. The twisting pitch P = 40 μm. The scale bar is 100 μm.

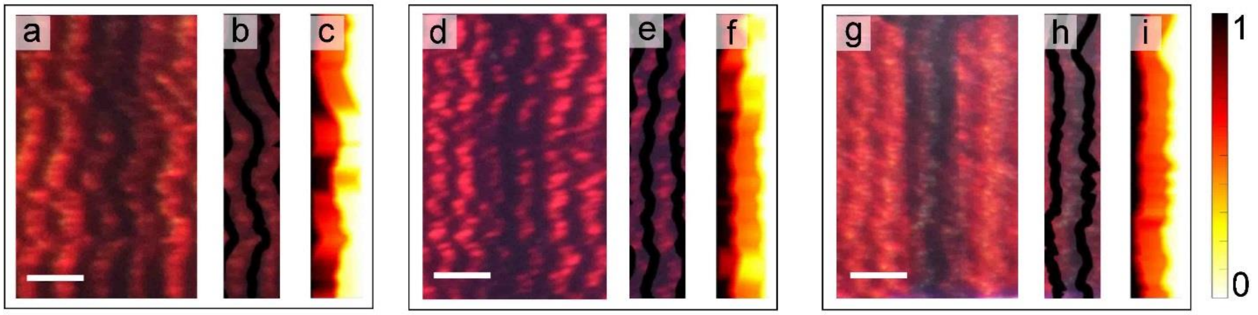

Figure S31. (a, d, g) Optical images of twisted crystals grown on OFET device. (b, e, h) Optical images combined with the boundary curves and (c, f, i) correspondingly simulated electrical potential distributions. (b) σ_x/P = 0.35 and (c) C/C_{ideal} = 0.27. (e) σ_x/P = 0.24 and (f) C/C_{ideal} = 0.23. (h) σ_x/P = 0.13 and (i) C/C_{ideal} = 0.22. The twisting pitch P = 60 μm. The scale bar is 100 μm.

References

(1) Shtukenberg, A. G.; Punin, Y. O.; Gunn, E.; Kahr, B. Spherulites. *Chem. Rev.* **2012**, *112*, 1805–1838.

(2) Fukuoka, E.; Makita, M.; Nakamura, Y. Glassy state of pharmaceuticals. V. Relaxation during cooling and heating of glass by differential scanning calorimetry. *Chem. Pharm. Bull.* **1991**, *39*, 2087–2090.

(3) Freudenthal, J. H.; Hollis, E.; Kahr, B. Imaging chiroptical artifacts. *Chirality* **2009**, *21*, 20–27.

(4) Cui, X.; Shtukenberg, A. G.; Freudenthal, J.; Nichols, S. M.; Khar, B. Circular birefringence of banded spherulites. *J. Am. Chem. Soc.* **2014**, *136*, 5481–5490.

(5) Cui, X.; Nichols, S. M.; Arteaga, O.; Freudenthal, J.; Paula, F.; Shtukenberg, A. G.; Kahr, B. Dichroism in helicoidal crystals. *J. Am. Chem. Soc.* **2016**, *138*, 12211–12218.

(6) Nečas, D.; Klapetek, P. Gwyddion: an Open-Source Software for SPM Data Analysis. *Cent. Eur. J. Phys*. **2012**, *10*, 181−188.

(7) Tan, M.; Jiang, W.; Matin, A. T.; Shtukenberg, A. G.; McKee, M.; Kahr, B. Polarized light through polycrystalline vaterite helicoids. *Chem. Commun*. **2020**, *56*, 7353-7356.

(8) Borchert, J. W.; Peng, B.; Letzjus, F.; Burghartz, J. N.; Chan, P. K. L.; Zojer, K.; Ludwigs, S.; Klauk, H. Small contact resistance and high-frequency operation of flexible low-voltage inverted coplanar organic transistors. *Nature Comm.* **2019**, *10*, 1119.

(9) Borchert, J. W.; Zschieschang, U.; Letzkus, F.; Giorgio, M.; Weitz, R. T.; Caironi, M.; Burghartz, J. N.; Ludwigs, S.; Klauk, H. Flexible low-voltage high-frequency organic thin-film transistors. *Sci. Adv.* **2020**, *6*, eaaz5156.

(10) Yang, Y.; Zhang, Y.; Hu, C. T. Sun, M.; Jeong, S.; Lee, S. S.; Shtukenberg, A. G.; Kahr, B. Transport in twisted crystalline charge transfer complexes. *Chem. Mater.* **2022**, *34*, 1778–1788.

(11) Bredas, J.-L.; Beljonne, D.; Coropceanu, V.; Cornil, J. Charge-transfer and energy-transfer processes in □-conjugated oligomers and polymers: A molecular picture. *Chem. Rev.* **2004**, *104*, 4971–5004.

(12) Du, J.; McCandless, G. T.; Stefan M. C. CCDC 2116030: Experimental Crystal Structure Determination, **2021**, DOI: 10.5517/ccdc.csd.cc290x19.

(13) Wen, S.-H.; Li, A.; Song, J.; Deng, W.-Q.; Han, K.-L.; Godard III W. A. First-principles investigation of anisotropic hole mobilities in organic semiconductors. *J. Phys. Chem. B* **2009**, *113*, 8813–8819.

(14) Yang, Y.; de Moraes, L. S.; Ruzie, C.; Schweicher, G.; Geerts, Y. H.; Kennedy, A. R.; Zhou, H.; Whittaker, S. J.; Lee, S. S.; Kahr, B.; Shtukenberg, A. G. Charge transport in twisted organic semiconductor crystals of modulated pitch. *Adv. Mater.* **2022**, *34*, 2203842.

(15) Zhu, L. Y.; Yi, Y. P.; Li, Y.; Kim, E. G.; Coropceanu, V.; Bredas, J.-L. Prediction of remarkable ambipolar charge-transport characteristics in organic mixed-stack charge-transfer crystals. *J. Am. Chem. Soc.* **2012**, *134*, 2340–2347.

(16) Marcus, R. A. Electrostatic free energy and other properties of states having nonequilibrium polarization. I. *J. Chem. Phys.***1956**, *24*, 979–989.

(17) Hush, N. S. Adiabatic rate processes at electrodes. I. Energy-charge relationships. *J. Chem. Phys.* **1958**, *28*, 962–972 (1958).

(18) Rigaku, O. D.; *CrysAlisPRO*, Rigaku Oxford Diffraction Ltd: Yarnton, Oxfordshire, England, **2017**.

(19) Kocak, O.; Duru, I. P.; Yavuz, I. Charge transfer and interface effects in co-assembled circular donor/acceptor complexes for organic photovoltaics. *Adv. Theor. Simul*. **2019**, *2*, 1800194.

(20) Coropceanu, V.; Cornil, J.; da Silva, D. A.; Olivier, Y.; Silbey, R.; Bredas, J.-L. Charge transport in organic semiconductors. *Chem. Rev.* **2007**, *107*, 926–952.

(21) Frisch, M. J.; Trucks, G. W.; Schlegel, H. B.; Scuseria, G. E.; Robb, M. A.; Cheeseman, J. R.; Scalmani, G.; Barone, V.; Petersson, G. A.; Nakatsuji, H.; Li, X.; Caricato, M.; Marenich, A. V.; Bloino, J.; Janesko, B. G.; Gomperts, R.; Mennucci, B.; Hratchian, H. P.; Ortiz, J. V.; Izmaylov, A. F.; Sonnenberg, J. L.; Williams-Young, D.; Ding, F.; Lipparini, F.; Egidi, F.; Goings, J.; Peng, B.; Petrone, A.; Henderson, T.; Ranasinghe, D.; Zakrzewski, V. G.; Gao, J.; Rega, N.; Zheng, G.; Liang, W.; Hada, M.; Ehara, M.; Toyota, K.; Fukuda, R.; Hasegawa, J.; Ishida, M.; Nakajima, T.; Honda, Y.; Kitao, O.; Nakai, H.; Vreven, T.; Throssell, K.; Montgomery, J. A. Jr.; Peralta, J. E.; Ogliaro, F.; Bearpark, M. J.; Heyd, J. J.; Brothers, E. N.; Kudin, K. N.; Staroverov, V. N.; Keith, T. A.; Kobayashi, R.; Normand, J.; Raghavachari, K.; Rendell, A. P.; Burant, J. C.; Iyengar, S. S.; Tomasi, J.; Cossi, M.; Millam, J. M.; Klene, M.; Adamo, C.; Cammi, R.; Ochterski, J. W.; Martin, R. L.; Morokuma, K.; Farkas, O.; Foresman, J. B.; Fox, D. J. Gaussian 16, Revision A.03, Gaussian Inc.: Wallingford, CT, **2016**.

(22) Shtukenberg, A. G.; Cui, X.; Freudenthal, J.; Gunn, E.; Camp, E.; Kahr, B. Twisted mannitol crystals establish homologous growth mechanisms for high-polymer and small-molecule ring-banded spherulites. *J. Am. Chem. Soc.* **2012**, *134*, 6354–6364.
